# Drosophila TDP-43 RNA-Binding Protein Facilitates Association of Sister Chromatid Cohesion Proteins with Genes, Enhancers and Polycomb Response Elements

**DOI:** 10.1371/journal.pgen.1006331

**Published:** 2016-09-23

**Authors:** Amanda Swain, Ziva Misulovin, Michelle Pherson, Maria Gause, Kathie Mihindukulasuriya, Ryan A Rickels, Ali Shilatifard, Dale Dorsett

**Affiliations:** 1 Edward A. Doisy Department of Biochemistry and Molecular Biology, Saint Louis University School of Medicine, Saint Louis, Missouri, United States of America; 2 Department of Biochemistry and Molecular Genetics, Northwestern Feinberg School of Medicine, Chicago, Illinois, United States of America; Geisel School of Medicine at Dartmouth, UNITED STATES

## Abstract

The cohesin protein complex mediates sister chromatid cohesion and participates in transcriptional control of genes that regulate growth and development. Substantial reduction of cohesin activity alters transcription of many genes without disrupting chromosome segregation. Drosophila Nipped-B protein loads cohesin onto chromosomes, and together Nipped-B and cohesin occupy essentially all active transcriptional enhancers and a large fraction of active genes. It is unknown why some active genes bind high levels of cohesin and some do not. Here we show that the TBPH and Lark RNA-binding proteins influence association of Nipped-B and cohesin with genes and gene regulatory sequences. In vitro, TBPH and Lark proteins specifically bind RNAs produced by genes occupied by Nipped-B and cohesin. By genomic chromatin immunoprecipitation these RNA-binding proteins also bind to chromosomes at cohesin-binding genes, enhancers, and Polycomb response elements (PREs). RNAi depletion reveals that TBPH facilitates association of Nipped-B and cohesin with genes and regulatory sequences. Lark reduces binding of Nipped-B and cohesin at many promoters and aids their association with several large enhancers. Conversely, Nipped-B facilitates TBPH and Lark association with genes and regulatory sequences, and interacts with TBPH and Lark in affinity chromatography and immunoprecipitation experiments. Blocking transcription does not ablate binding of Nipped-B and the RNA-binding proteins to chromosomes, indicating transcription is not required to maintain binding once established. These findings demonstrate that RNA-binding proteins help govern association of sister chromatid cohesion proteins with genes and enhancers.

## Author Summary

The cohesin protein complex binds chromosomes to facilitate their proper division into two daughter cells when a cell divides. Cohesin and the Nipped-B protein that loads cohesin onto chromosomes also bind to genes and regions that regulate genes to ensure that genes produce proper amounts of RNA. Nipped-B and cohesin preferentially bind a subset of genes important for growth and development, and small changes in cohesin activity cause severe birth defects. Why cohesin binds some genes and not others is unknown. We tested the idea that proteins that specifically bind the RNA sequences being produced by genes may help determine which genes bind cohesin. We find that the Drosophila TBPH and Lark RNA-binding proteins bind RNA produced by genes that bind Nipped-B and cohesin. They also bind to these genes on chromosomes and the chromosomal regions that regulate them. TBPH ensures that Nipped-B and cohesin are present at high levels on regulatory regions and genes. Lark inhibits Nipped-B and cohesin binding to some genes and increases their binding to some regulatory regions. Nipped-B interacts with both RNA-binding proteins and promotes their binding to genes. These findings show that RNA-binding proteins help determine which genes bind Nipped-B and cohesin.

## Introduction

The cohesin complex plays crucial roles in sister chromatid cohesion, chromosome segregation, DNA repair, and gene transcription [1–3]. Cohesin is composed of the SMC1 (Flybase FBgn0040283) and SMC3 (Flybase FBgn0015615) structural maintenance of chromosome proteins, the Rad21 (verthandi, Flybase FBgn0260987) kleisin protein, and Stromalin (SA, Flybase FBgn0020616), which form a ring-shaped complex that encircles chromosomes. Cohesin is loaded topologically onto chromosomes by the kollerin protein complex consisting of Nipped-B (Flybase FBgn0026401) and Mau-2 (CG4203, Flybase FBgn0038300) [4].

Organismal development is exquisitely sensitive to changes in Nipped-B and cohesin activity. In Drosophila, mice and humans, mutations that reduce Nipped-B dosage by less than 30%, or that slightly alter cohesin subunit structure, cause reduced growth, structural abnormalities and intellectual deficits [2, 5–7]. In humans, these genetic diseases are collectively called cohesinopathies, and include Cornelia de Lange Syndrome (CdLS). More than half the cases of CdLS are caused by heterozygous loss-of-function mutations in the *NIPBL* ortholog of *Nipped-B* [8, 9]. CdLS caused by *NIPBL* mutations generally displays more severe physical alterations, and more mild CdLS cases are often caused by dominant missense mutations in the *SMC1A* or *SMC3* cohesin subunit genes [6]. A small number of CdLS cases are caused by dominant loss-of-function mutations in *HDAC8*, whose protein product recycles acetylated SMC3 [10]. Heterozygous *Nipped-B*, *NIPBL*, or *Nipbl* mutations do not measurably alter sister chromatid cohesion or chromosome segregation, and thus the diverse developmental deficits likely reflect hundreds of changes in gene expression [7, 11–14].

Nipped-B and cohesin participate in the control of gene transcription via multiple mechanisms. For instance, cohesin facilitates looping interactions between genes and distant transcriptional enhancers, and functionally interacts with Polycomb Group (PcG) silencing proteins to modify RNA polymerase activity at active and silenced genes [2, 5, 15]. Genome-wide chromatin immunoprecipitation (ChIP) shows that Nipped-B and cohesin occupy essentially all active transcriptional enhancers, all active Polycomb Response Elements (PREs) essential for epigenetic silencing, and a subset of active gene promoters [16–18]. The subset of active genes occupied and regulated by Nipped-B and cohesin is enriched for genes that control growth and development, consistent with the phenotypes of *Nipped-B* mutants [7].

It is unknown why cohesin preferentially associates with some active genes and not others. In *Drosophila*, cohesin binding genes share certain common features, including the presence of transcriptionally-paused RNA polymerase (Pol II) just downstream of the transcription start site [17, 19]. However, depletion of the NELF (Negative Elongation Factor) and DSIF (DRB-sensitivity inducting factor) complexes that establish pausing does not reduce cohesin occupancy at these promoters [19]. Although cohesin depletion decreases or increases how frequently paused Pol II enters into active elongation at many genes, the levels of paused Pol II at the promoters do not appreciably change, indicating that Nipped-B and cohesin act downstream of pausing establishment to modulate transition of paused polymerase to elongation [17, 19].

Prior studies revealed that the active genes occupied by Nipped-B and cohesin are highly enriched for TG dinucleotide repeats downstream of the transcription start site (TSS) in the non-template strand, relative to active genes that don’t bind cohesin [19]. This raised the possibility that RNA sequences and RNA binding proteins could influence association of Nipped-B and cohesin with these genes. Here we show that Nipped-B interacts with two RNA-binding proteins, TBPH (Tar DNA binding protein homolog, TDP-43, Flybase FBgn0025790) and Lark (Flybase FBgn0011640). TBPH and Lark both co-localize on chromosomes with Nipped-B and cohesin at active genes, enhancers, and Polycomb Response Elements (PREs). TBPH binds RNAs containing UG repeats, and Lark preferentially binds RNAs produced by genes that bind Nipped-B. Depletion of TBPH globally reduces Nipped-B and cohesin association with active genes and regulatory sequences. In contrast, Lark depletion increases their levels at many promoters, but reduces their association with some large “super-enhancers”. Conversely, Nipped-B depletion reduces association of both Lark and TBPH with chromosomes. Unexpectedly, blocking transcription with triptolide does not ablate binding of Nipped-B, TBPH and Lark to gene regulatory elements, indicating that continued transcription is not required to maintain their binding once it has been established. We posit that TBPH binding to pioneer nascent transcripts recruits Nipped-B to initiate cohesin and Lark association with specific genes, and that protein interactions maintain their association with chromosomes in the absence of continued transcription. These findings indicate that RNA-binding proteins help determine which genes bind Nipped-B and cohesin, and raise the possibility that Nipped-B can impact RNA processing by facilitating association of RNA-binding proteins with genes.

## Results

### The TBPH RNA-binding protein associates with Nipped-B and cohesin-binding genes, enhancers and Polycomb response elements (PREs) on chromosomes

In a prior quantitative sequence analysis of 506 active genes that bind Nipped-B and cohesin at high levels, and 1,040 active genes that do not, consecutive repeats of the TG dinucleotide sequence were found to be greatly enriched above random expectation within 50 to 800 bp downstream of the transcription start sites of the cohesin-binding genes, but not in the genes that don’t bind cohesin (see reference [19] for details). The TG repeats are exclusively in the non-template strand and intron sequences, and thus produce nascent RNAs that contain UG repeats. TBPH (TAR DNA binding protein homolog) is the Drosophila homolog of mammalian TDP-43 that specifically binds UG repeats in RNA and regulates RNA processing [20, 21]. TBPH is essential for Drosophila development, with lethality occurring during pupal development [22].

**Fig 1:**
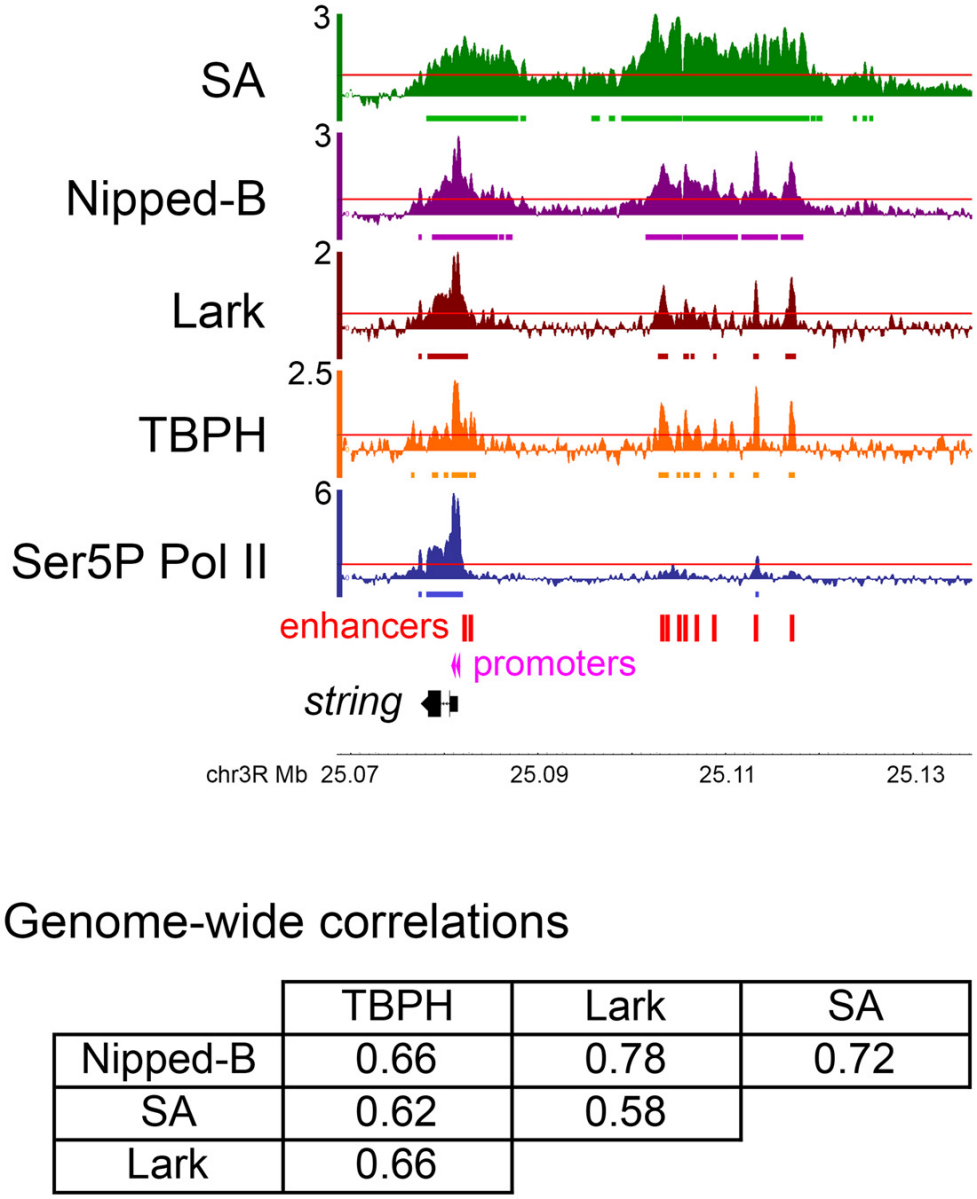
Co-occupancy of genes and regulatory sequences by Nipped-B, cohesin, and the TBPH and Lark RNA-binding proteins in BG3 cells. The genome browser tracks show the log2 ChIP-seq enrichment for cohesin (SA, green), Nipped-B (purple), Lark (brown), TBPH (orange) and Ser5P Pol II (transcriptionally-engaged paused and elongating Pol II, blue) at the *string* (*cdc25*) gene, and its upstream enhancers (red boxes). The two active *string* transcription start sites detected by PRO-seq [17] are indicated by pink arrowheads. Genome-wide Pearson correlation coefficients between Nipped-B, SA, TBPH and Lark ChIP-seq enrichment are in the table below the genome browser panel. The ChIP-seq enrichment for SA, Nipped-B and Ser5P Pol II are the average of two independent biological replicates (independent cell cultures, chromatin preparations and immunoprecipitation). The Lark ChIP-seq data is the average of five biological replicates, and the TBPH ChIP-seq is the average of four biological replicates. Each replicate was sequenced to ~10X genome coverage and normalized to input chromatin to calculate sequence enrichment in 250 bp sliding windows positioned 50 bp apart [24]. This procedure gives smoothened enrichment values at data points spaced 50 bp apart. As illustrated by the browser tracks, this method detects occupancy both in broad regions and in narrow peaks. It provides sensitivity equivalent to ChIP-chip for Nipped-B and cohesin [24]. Red lines in each browser track indicate the 95^th^ percentile for enrichment for that protein, and the bars beneath each track show where enrichment is >95^th^ percentile over regions >300 bp in length (>6 consecutive data points).

We hypothesized that TBPH binding to nascent RNA transcripts could recruit Nipped-B and cohesin to active genes. This predicts that TBPH will bind to chromosomes specifically at cohesin-binding genes. We tested this prediction using genome-wide chromatin immunoprecipitation (ChIP-seq) for TBPH in cultured BG3 cells derived from larval central nervous system. This revealed that TBPH binds chromosomes in a pattern very similar to Nipped-B and cohesin (Fig 1). TBPH binding correlates strongly with Nipped-B, with a genome-wide Pearson correlation coefficient of 0.66, similar to the Nipped-B—SA cohesin subunit correlation of 0.72 (Fig 1). The TBPH—SA correlation (0.62) is also high (Fig 1).

The TBPH antibodies used for ChIP-seq were validated by RNAi depletion and western blotting (S1 Fig) Validation of the Nipped-B and SA antibodies was described previously [23]. Four biological replicate ChIP-seq experiments were performed for TBPH, with each replicate sequenced to ~10X genome coverage, and normalized to input chromatin sequenced to >40X genome coverage. Two biological replicate ChIP-seq experiments were performed for Nipped-B, and three for SA, whose genome-wide binding in BG3 cells were previously mapped by ChIP-chip [23]. The ChIP-seq patterns closely match those previously mapped by ChIP-chip [23, 24].

**Fig 2:**
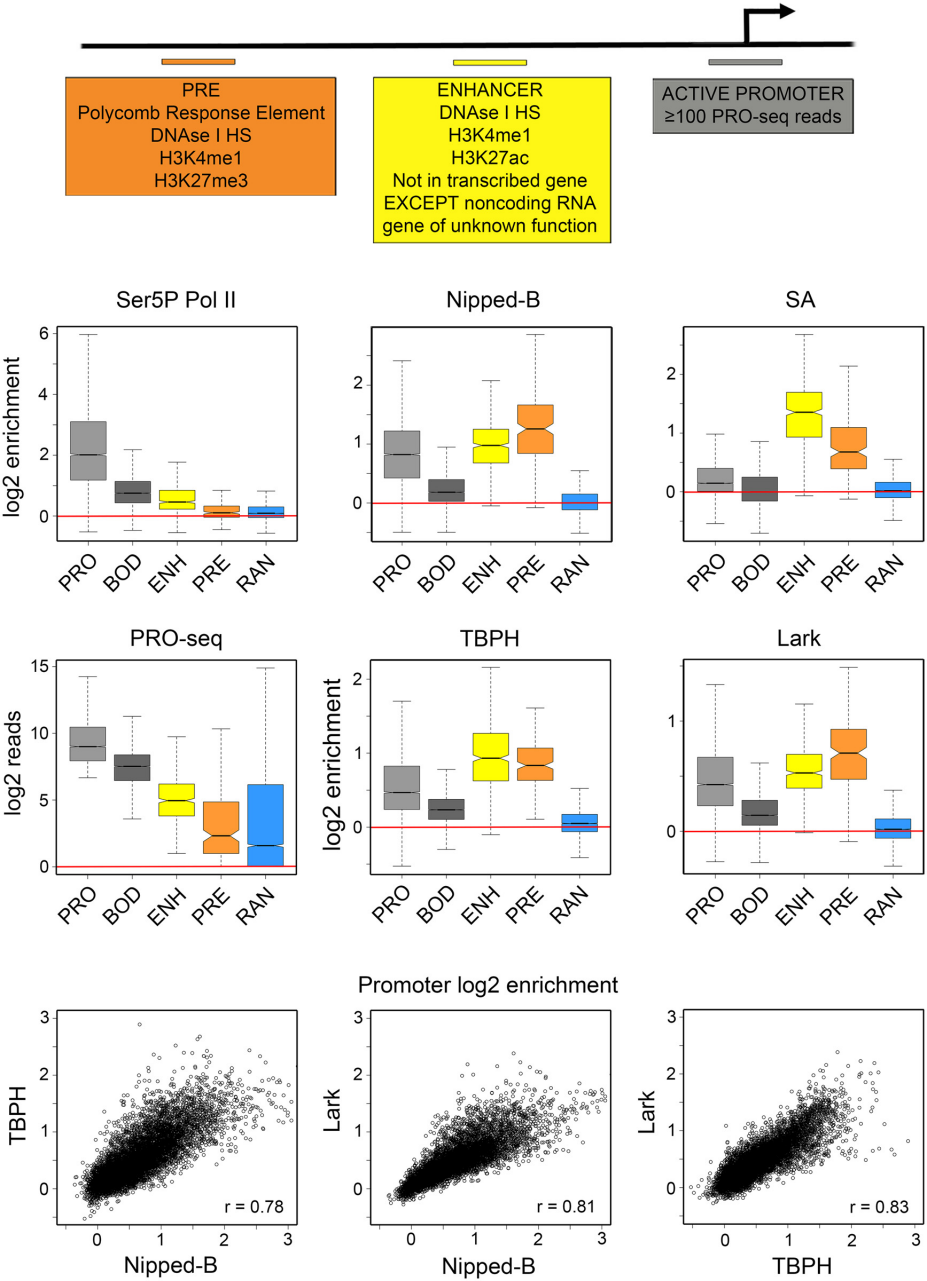
TBPH and Lark bind enhancers, PREs, and the same active as Nipped-B and cohesin in BG3 cells. The top diagram indicates how active promoters, extragenic enhancers, and PREs were defined for the analyses shown in the box and dot plots. Active promoters (light gray, PRO) are defined as 500 bp regions surrounding the transcription start sites (-250 to +250) that have at least 100 sequence reads in the published PRO-seq data [17] (GEO accession GSE42397). Excluding those that fall in heterochromatin, where ChIP-seq shows poor coverage, there are 7,389 active promoters. Enhancers (ENH) are defined as 500 bp elements centered at the peaks of DNase I hypersensitive sites that show H3K4me1 and H3K27ac histone modifications. This predicts 2,353 active enhancers, many of which correspond with known enhancers, such as those drive expression of *string* (Fig 1). The analysis performed here used all 523 extragenic enhancers in euchromatin positioned at least 500 bp outside of an actively transcribed gene (except non-coding RNAs of unknown function) [17]. Polycomb Response Elements (PREs) are defined as DNase I hypersensitive sites that show H3K4me1 and H3K27me3 histone modifications [18]. This identifies 195 PREs, including all known active PREs in BG3 cells, such as those silencing the *vestigial* gene (S2 Fig). As controls we also quantified occupancy of 7,389 gene body 500 bp segments starting 500 nt downstream of all active promoters (BOD, dark gray), and 6,892 randomly-positioned 500 bp regions (RAN, blue). The box plots show the log2 of the average ChIP-seq enrichment of all data points (usually 10) falling within each 500 bp region. An example of the method used to calculate the average enrichment at promoters is shown in S4 Fig. Red lines indicate where there is no ChIP-seq enrichment (log2 = 0, enrichment = 1). The PRO-seq box plots show the log2 of the number of sequence reads from both strands that fall within each 500 bp region as a measure of transcription. The dot plots below show the log2 average enrichment values at all active promoters for Nipped-B vs. TBPH, Nipped-B vs. Lark, and TBPH vs. Lark. Each point represents one of the 7,389 active promoters.

The strong correspondence in binding of TBPH with Nipped-B and cohesin is illustrated at the *string* (*cdc25*) gene, where Nipped-B and cohesin bind to the gene and strikingly, also to the upstream large “super-enhancer” region (Fig 1). The locations of the multiple enhancers in this region were predicted using modENCODE DNaseI hypersensitivity and histone modification data [25] as previously described [17, 18] (Fig 2) and are all contained within regions shown to drive *string* expression in vivo [26]. Binding of TBPH to the enhancer region was unexpected, because it shows only low levels of bidirectional transcription by precision global run-on sequencing (PRO-seq) [17]. As detailed below, TBPH occupies essentially all active enhancers. Even more surprisingly, TBPH also co-occupies PREs (Polycomb response elements) with Nipped-B and cohesin, despite even lower transcription levels than enhancers. S2 Fig shows binding of Nipped-B and TBPH at PREs that silence the *vestigial* gene in BG3 cells.

### The Lark RNA-binding protein also associates with cohesin-binding genes, enhancers and PREs on chromosomes

TBPH was predicted to associate with cohesin-binding genes because TBPH was known to bind to UG repeats in RNA. We also considered the possibility that other RNA-binding proteins might preferentially recognize nascent RNAs from cohesin-binding genes. As described in S3 Fig, RNA affinity chromatography and mass spectrometry identified Lark as a likely candidate for such a protein. Strikingly, our analysis of the RNAs that immunoprecipitated with Lark in a prior study [27] revealed that essentially all are produced by cohesin-binding genes (p = 2.5E-58, Fisher’s exact test, S3 Fig). Lark is an ortholog of mammalian RBM4 proteins, which are involved in RNA splicing and exon definition [28] and homozygous *lark* mutations are lethal early in development. We thus also conducted ChIP-seq for Lark to determine if it also associates specifically with cohesin-binding genes on chromosomes.

This revealed that Lark, like TBPH, also binds chromosomes in a pattern very similar to Nipped-B and cohesin (Fig 1). Lark genome-wide binding correlates well with Nipped-B (0.78), SA (0.58) and TBPH (0.66) (Fig 1). Like TBPH, Lark also binds to the *string* enhancers (Fig 1) and *vestigial* PREs (S2 Fig). We validated the Lark antibodies by RNAi depletion and western blotting (S1 Fig) and performed five biological replicate ChIP-seq experiments.

### TBPH and Lark occupy the same gene regulatory sequences as Nipped-B and cohesin

We performed quantitative analysis of the ChIP-seq data to more accurately determine the extent to which the presence of TBPH and Lark at gene regulatory sequences corresponds with Nipped-B, cohesin and transcription. For this we defined active promoters, extragenic enhancers and PREs as 500 bp elements as described in prior work [17,18] and outlined in Fig 2. Our laboratory previously showed that Nipped-B occupies roughly a third of the 7,389 active euchromatic gene promoters predicted by PRO-seq and essentially all enhancers and PREs using a statistical threshold (p ≤ 10E-4) with ChIP-chip data to call binding [17, 18]. Although we predict 2,353 active enhancers, for the analysis described here we used only 523 extragenic enhancers, including the ten associated with *string* (Fig 1) that are positioned more than 500 bp outside of transcribed genes. Restricting the analysis to enhancers that are not located in transcribed genes allows us to assess the potential role of transcription in binding of TBPH and Lark to enhancers. Although we predict only 195 active PREs, prior studies have shown that all large H3K27me3 domains are associated with one more of these PREs, which bind the PRC1 Polycomb complex, and many of which overlap known PREs [18]. All PREs were used for analysis.

We averaged the enrichment values at all ten ChIP-seq data points that fall within each 500 bp regulatory sequence as illustrated in S4 Fig. As controls, we also averaged enrichment over 500 bp segments of active gene bodies, starting 500 bp downstream of the transcription start site (+500 to +1000) and 6,892 random locations (Fig 2). We quantified PRO-seq levels and Pol II ChIP-seq enrichment to gauge transcriptional activity in the regulatory regions (Fig 2). For Pol II we performed two biological replicate ChIP-seq experiments for RNA polymerase II phosphorylated at the serine 5 residues in the heptad repeats of the Rpb1 C terminal domain (Ser5P Pol II). The Ser5P Pol II antibody recognizes Pol II that has initiated transcription, but remains paused just downstream of the transcription start site, and Pol II that is elongating in gene bodies, as illustrated at the *string* gene (Fig 1).

As the boxplots in Fig 2 illustrate, the median ChIP-seq enrichment at random control positions (RAN) in the genome is close to 1 (log2 = 0) for all proteins, indicating little or no binding. All proteins show significant occupancy at 7,389 active gene promoters (PRO) and lower levels in the bodies (BOD) of these genes. Ser5P Pol II shows a median 4-fold enrichment at active promoters, and a 1.4-fold median enrichment at extragenic enhancers, and very little enrichment (<1.1-fold) at PREs. Although low, the enrichment of Ser5P Pol II at extragenic enhancers is significant relative to the levels at random sites (p = 2.5E-50, t test) and exceeds the 95^th^ percentile for enrichment over regions >300 bp in length at many enhancers, including one associated with *string* (Fig 1).

Enhancer-promoter looping can permit Pol II in the promoter region to crosslink to the enhancer and thus we cannot know if the Ser5P Pol II detected at enhancers is transcribing the enhancers or the gene. However, the Ser5P Pol II enrichment at promoters, enhancers, and PREs correlates with transcription measured by PRO-seq (Fig 2). The Pearson correlation coefficient between the Ser5P Pol II ChIP-seq and PRO-seq reads is 0.70 at promoters and 0.54 at enhancers, indicating that at least some Pol II is transcriptionally-engaged at enhancers.

Importantly, unlike Ser5P Pol II, Nipped-B, cohesin (SA), TBPH and Lark all show higher median occupancy at extragenic enhancers and PREs than at active promoters (Fig 2). For Nipped-B and cohesin, the higher median occupancy at enhancers and PREs is consistent with the prior finding that Nipped-B and cohesin occupy essentially all active enhancers and PRES, but only a third of active gene promoters, when binding is called using a statistical threshold with ChIP-chip data [17, 18]. The dot plots in Fig 2 show that TBPH and Lark occupancy at promoters correlate strongly with each other and Nipped-B (r = 0.8). Thus TBPH and Lark bind to the same active genes as Nipped-B and cohesin, consistent with the hypothesis that they may influence binding of Nipped-B and cohesin to active genes.

The finding that TBPH and Lark occupy the vast majority of active enhancers and PREs was unexpected because enhancers and PREs are transcribed at substantially lower levels than promoters, and thus produce lower levels of nascent RNA transcripts. By PRO-seq, measuring transcription of both strands, the median transcription level at extragenic enhancers is 16.5-fold less than at promoters, and more than 100-fold less at PREs (Fig 2). This suggests that interactions of TBPH and Lark with proteins or DNA, and not just interactions with nascent RNA transcripts, may contribute to their binding to gene regulatory sequences.

We conducted ChIP-seq of Nipped-B, TBPH and Lark in 3^rd^ instar larval wing discs to confirm that the binding pattern of the RNA proteins to regulatory sequences occurs in vivo and is not unique to cultured BG3 cells. Our laboratory previously reported ChIP-chip for Nipped-B, cohesin and several other proteins in 3^rd^ instar wing discs, showing that they are similar to the patterns observed in multiple cultured cell lines [18]. S5 Fig shows that the genome-wide correlation between TBPH and Nipped-B is 0.65 and the Lark correlation to Nipped-B is 0.75 in developing wings. The binding pattern at the *string* and *Wrinkled* genes in wing discs are similar to those observed in BG3 cells (S5 Fig).

### TBPH globally facilitates Nipped-B and cohesin occupancy of genes and regulatory sequences

Using genomic ChIP, we found that depletion of TBPH by RNAi in BG3 cells reduces Nipped-B and cohesin chromosome occupancy genome-wide, with significant global reductions at gene regulatory sequences. Nipped-B and SA (cohesin) ChIP-seq was performed with two biological replicates for TBPH depletion, which showed close statistical correlation with each other and were averaged for comparison to the averaged mock-treated control ChIP-seq replicates shown in Fig 1. TBPH depletion by greater than 90% (S1 Fig) did not detectably alter the rate of cell division, cell morphology, or decrease cell viability, indicating little or no effect on progression of the cell cycle. Prior studies show that stopping cell division and inducing differentiation of BG3 cells by depletion of PRC1 subunits does not globally alter cohesin binding to chromosomes [18] and thus TBPH depletion is unlikely to alter Nipped-B and cohesin binding through effects on the cell cycle. TBPH depletion also did not appreciably alter the levels of Nipped-B, or the Rad21 and SA cohesin subunit proteins (S1 Fig).

**Fig 3:**
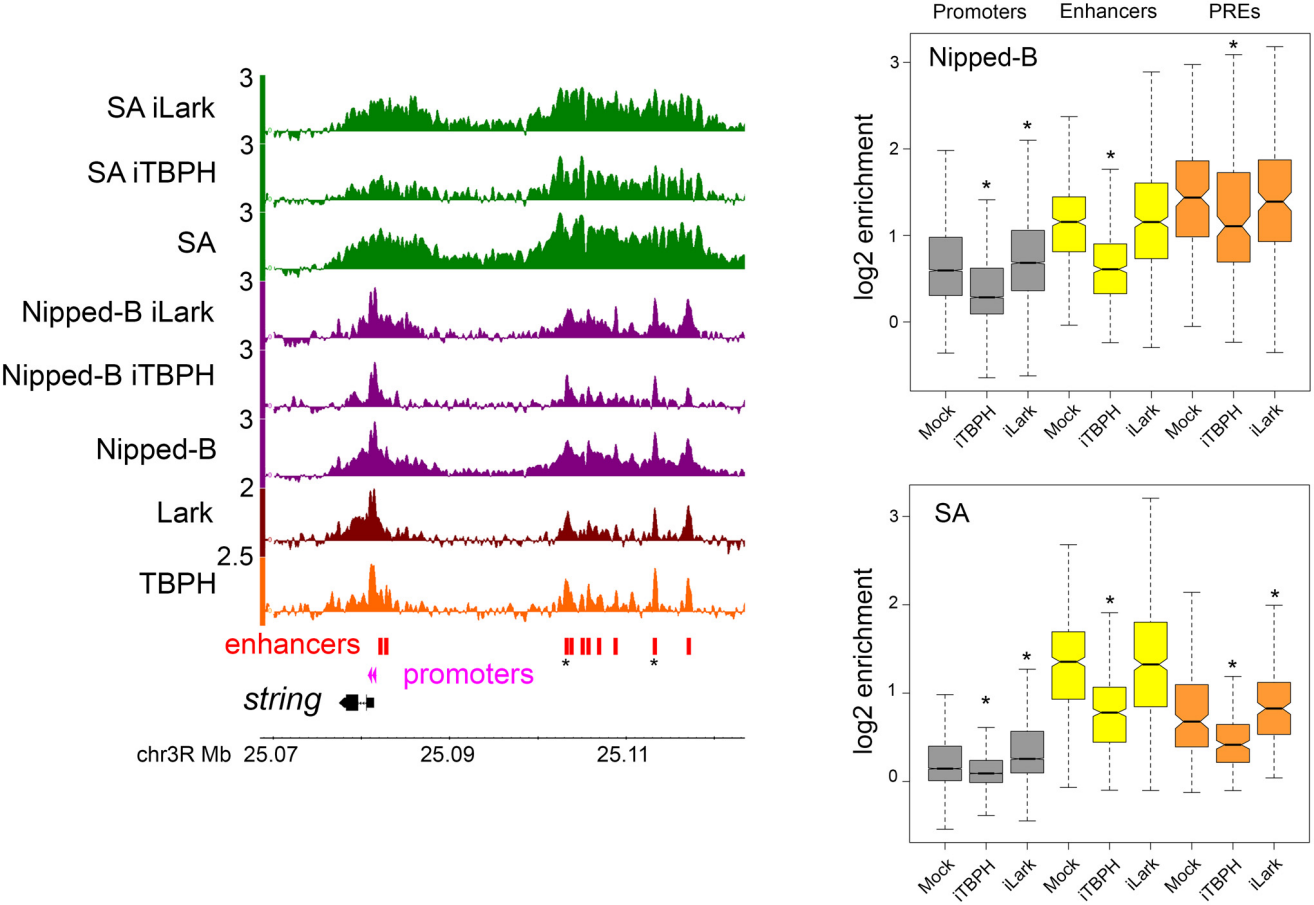
TBPH and Lark depletion alter Nipped-B and cohesin occupancy at gene regulatory sequences in BG3 cells. The browser tracks show the log2 enrichment for TBPH, Lark, Nipped-B, SA, and Nipped-B and SA after RNAi depletion of TBPH (iTBPH) and Lark (iLark) for the *string* gene and its upstream enhancers. The *string* enhancers that are most resistant to decreases in Nipped-B and SA occupancy upon TBPH depletion are marked with asterisks. The tracks shown are the average of two to five independent biological replicates. The boxplots show the distributions of the average log2 enrichment for all active promoters (gray), extragenic enhancers (yellow) and PREs (orange) in mock-treated (Mock) cells, and cells in which TBPH (iTBPH) or Lark (iLark) have been depleted by RNAi for four to five days. Gene regulatory elements are defined as shown in Fig 2. Asterisks indicate distributions that differ significantly from the mock control using a paired t test. The p values are given in the main text. A paired t test was used because each regulatory sequence is matched with itself in the control and depleted cells and the distributions are close to normal. All comparisons indicated as significant are also significant using the non-parametric Wilcoxon signed rank test. Plots of the values for all individual regulatory sequences in mock control versus the depleted cells are shown in S6 Fig.

The boxplots in Fig 3 and dot plots in S6 Fig summarize the global effects of TBPH depletion on Nipped-B and cohesin occupancy at active promoters, extragenic enhancers and PREs. TBPH depletion reduces the Nipped-B and SA occupancy of the majority of promoters, enhancers and PREs (Fig 3 and S6 Fig). The reductions in Nipped-B ChIP-seq enrichment are significant at promoters (p = 0, paired t test) enhancers (p = 1.8E-159) and PREs (p = 2.9E-20). ChIP-seq reductions are also significant for the SA cohesin subunit at promoters (p = 0) enhancers (p = 9.5E-205) and PREs (p = 6.4E-45). As illustrated by the dot plots in S6 Fig many reductions in Nipped-B and SA ChIP-seq enrichment are on the order of 2-fold. It is also apparent from the dot plots that the vast majority of promoters, enhancers and PREs show reductions and that the magnitudes of the reductions vary between different elements. For instance, Nipped-B is reduced from 1.2 to 1.9-fold at different *string* enhancers (Fig 3). The reduction in SA enrichment at the ten *string* enhancers ranges from 1.2 to 2.5-fold. We conclude that TBPH facilitates the Nipped-B and cohesin occupancy of most gene regulatory sequences.

### Lark modifies Nipped-B and cohesin binding in multiple ways

Lark depletion has complex effects on Nipped-B and cohesin binding. Depletion of Lark by greater than 90% (S1 Fig) did not decrease cell viability, but modestly reduced the cell division rate by <10%, and induced a fraction of cells to form neuron-like processes. There were no measurable effects on the levels of Nipped-B, cohesin subunits or TBPH protein levels. There are significant increases in the median ChIP-seq enrichment levels for Nipped-B (p = 5.5E-231) and SA (p = 0) at active promoters (Fig 3). The dot plots in S6 Fig show that the increases occur at the majority of active promoters, with a minority showing decreases or no change. Lark depletion causes small increases in Nipped-B ChIP-seq enrichment at enhancers (p = 0.050) and PREs (p = 0.075). Given their modest nature and borderline statistical significance we do not think these represent significant global effects, but likely reflect small effects on Nipped-B occupancy at a minority of specific enhancers and PREs. There is no significant overall change in SA enrichment at enhancers (p = 0.22) but a small overall increase at PREs (p = 1.3E-7) (Fig 3 and S6 Fig). Thus, Lark has a modest overall negative influence on Nipped-B and cohesin binding to promoters, and a mild negative influence on cohesin occupancy at a majority of PREs.

Although the global effect of Lark on Nipped-B and SA at enhancers is minimal, there are several individual enhancers at which Lark depletion alters cohesin levels, with increases approaching 2-fold at some, and decreases exceeding 2-fold at others, as revealed by the dot plots in S6 Fig. Enhancers showing decreases tend to be clustered together in “super-enhancers” such as those at *string* (Fig 3) *Wrinkled* (*hid*) *diminutive* (*myc*) and *cut*. There is little reduction in Nipped-B at the ten *string* enhancers upon Lark depletion but a clear reduction in SA. There is also a reduction in SA near the *string* promoters, opposite to the general trend of increases at the majority of promoters.

There also more complex changes in Nipped-B and cohesin distribution at individual genes upon Lark depletion. For instance, similar to cases at other super-enhancers, Nipped-B and cohesin decrease over the 20 kb upstream enhancer region of the *headcase* gene, but also increase at multiple locations within the 85 kb transcribed region (S7 Fig). Similar increases in Nipped-B and cohesin in gene bodies upon Lark depletion occur at other genes such as *tramtrack*.

Thus Lark depletion causes multiple types of changes in Nipped-B and cohesin binding, with the primary trends being increases in cohesin levels at promoters and PREs, and decreases in cohesin at multiple super-enhancers. Because these effects are generally smaller for Nipped-B than for cohesin, we posit that Lark might inhibit cohesin loading by Nipped-B, or facilitate cohesin removal. In contrast to TBPH depletion, which globally reduces Nipped-B and cohesin binding, the effects observed with Lark are more modest and less global. Because Lark depletion noticeably slows cell proliferation and alters cell morphology while TBPH depletion does not, the modest effects of Lark depletion confirms that the strong global effects of TBPH on Nipped-B and cohesin binding are not likely caused by changes in cell cycle progression.

### Nipped-B facilitates binding of TBPH and Lark to genes and regulatory sequences

The finding that TBPH and Lark co-localize with Nipped-B and cohesin at enhancers and PREs that are transcribed at low levels led us to consider the possibility that Nipped-B or cohesin might facilitate their binding to these regions. To test this idea we performed ChIP-seq for TBPH and Lark in BG3 cells depleted for Nipped-B. We averaged four independent biological replicates for TBPH and two for Lark. As previously reported [16] substantial Nipped-B depletion (S1 Fig) slightly slows cell division with a mild G2 delay, but does not measurably reduce sister chromatid cohesion or alter chromosome segregation. It also does not increase cell death or noticeably affect cell morphology. Levels of the Rad21 and SA cohesin subunits are modestly reduced upon Nipped-B depletion but TBPH and Lark levels are not appreciably altered (S1 Fig).

**Fig 4:**
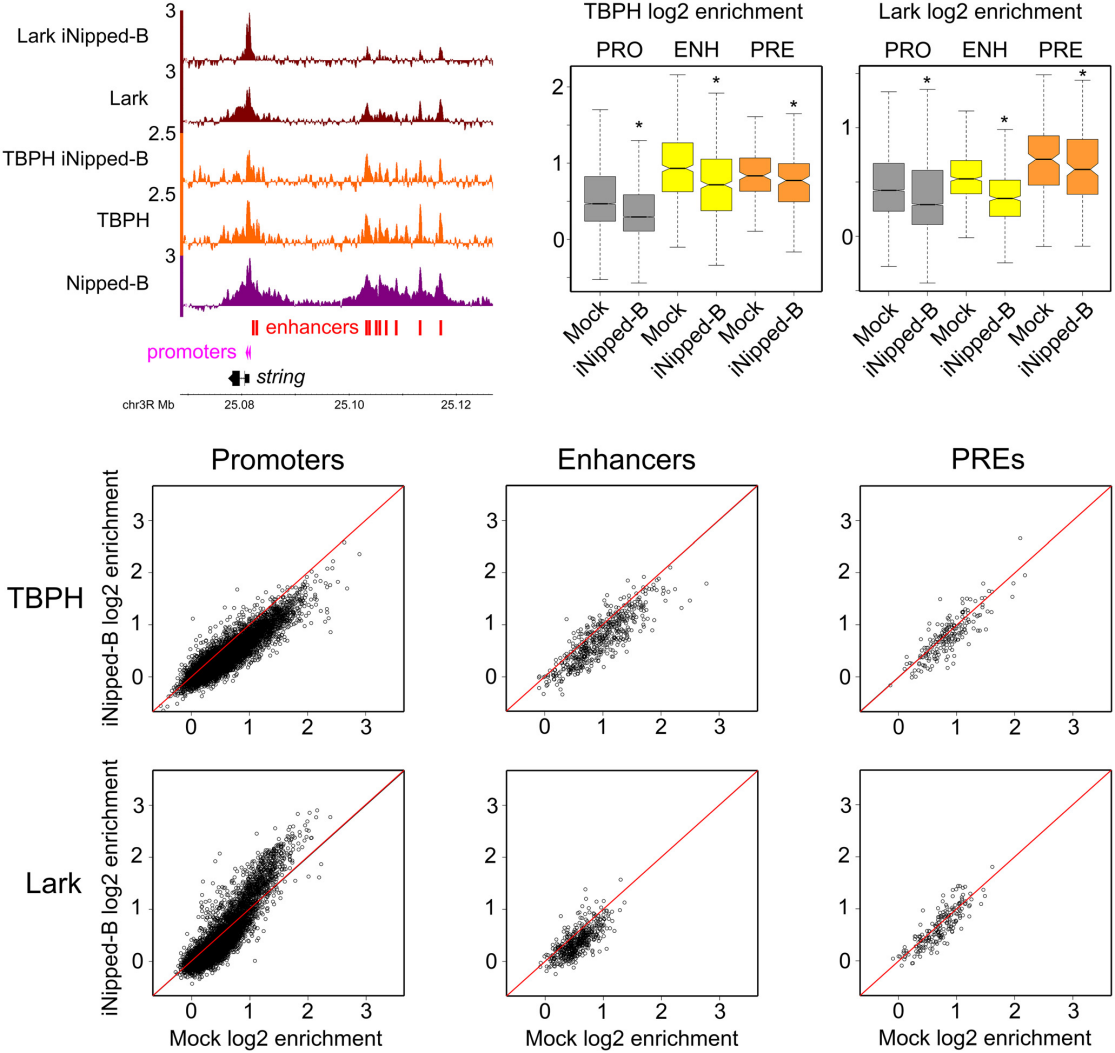
Nipped-B facilitates binding of TBPH and Lark to gene regulatory sequences in BG3 cells. The browser view at the upper left shows log2 ChIP-seq enrichment tracks for Nipped-B in control cells, and for TBPH and Lark in control cells and cells depleted for Nipped-B (iNipped-B) at the *string* gene and its enhancers. The boxplots at the upper right show the distributions of ChIP-seq enrichment for TBPH and Lark at active promoters (PRO) extragenic enhancers (ENH) and PREs (PRE) in control (Mock) cells and cells depleted for Nipped-B (iNipped-B). Four biological replicates were averaged for the TBPH ChIP-seq and two for Lark ChIP-seq in Nipped-B depleted cells. Distributions that differ significantly from the Mock control by the paired t test are marked with asterisks, and the p values are given in the main text. All marked comparisons are also significant by the Wilcoxon signed rank test. For each regulatory sequence, the log2 enrichment values in Mock control cells (Mock log2 enrichment) are plotted against the enrichment values in cells depleted for Nipped-B (iNipped-B log2 enrichment) in the dot plots.

Nipped-B depletion causes significant reduction in the ChIP-seq enrichment of TBPH at promoters (p = 0) enhancers (p = 5.4E-71) and PREs (p = 5.3E-6) as illustrated by the boxplots in Fig 4. Although the reductions are small, they occur at the majority of regulatory sequences that bind TBPH as shown by the Fig 4 dot plots. The decrease in TBPH at promoters and enhancers is illustrated by the changes at the *string* gene shown in Fig 4. These results are consistent with the idea that Nipped-B or cohesin can recruit or stabilize TBPH binding at gene regulatory sequences.

Nipped-B depletion also reduces ChIP-seq enrichment for Lark at promoters (p = 2.4E-64) enhancers (p = 5.6E-76) and PREs (p = 1.9E-4) as shown in the Fig 4 boxplots. The dot plots show that decreases occur at most enhancers and PREs, but the effect on Lark occupancy at promoters is bimodal. Promoters that start with lower levels of Lark in control cells show decreases upon Nipped-B depletion, but promoters with higher levels show increased occupancy. This is observed at *string*, where a decrease in Lark at the enhancers is accompanied by an increase at the promoters (Fig 4). We theorize that Nipped-B facilitates Lark binding to poorly-transcribed enhancers and PREs and those promoters whose nascent RNAs have lower affinity for Lark. Nipped-B depletion releases Lark from these regulatory sequences, making it available to bind at the promoters with nascent RNAs that have higher affinity for Lark.

### Inhibiting transcription does not ablate TBPH or Lark binding to genes and their regulatory sequences

We reasoned that if nascent RNA transcripts recruit TBPH to active genes, and TBPH then facilitates binding of Nipped-B and cohesin, then blocking transcription initiation might decrease binding of the RNA-binding and sister chromatid cohesion proteins to active genes. We find, however, that with the exception of Lark at promoters, blocking transcription does not ablate binding of Nipped-B or the RNA-binding proteins to gene regulatory sequences.

To block transcription we used triptolide to inhibit the TFIIH transcription initiation factor [29, 30]. We made chromatin after 1, 2 and 4 hours of triptolide treatment, and conducted ChIP-seq for Ser5P Pol II, Nipped-B, TBPH and Lark. The cells remained viable through 4 hours of treatment, with only a small fraction of cells starting to die at 4 hours as observed by trypan blue staining. Triptolide treatment for up to 4 hours did not measurably alter the levels of Nipped-B, TBPH or Lark (S8 Fig).

**Fig 5:**
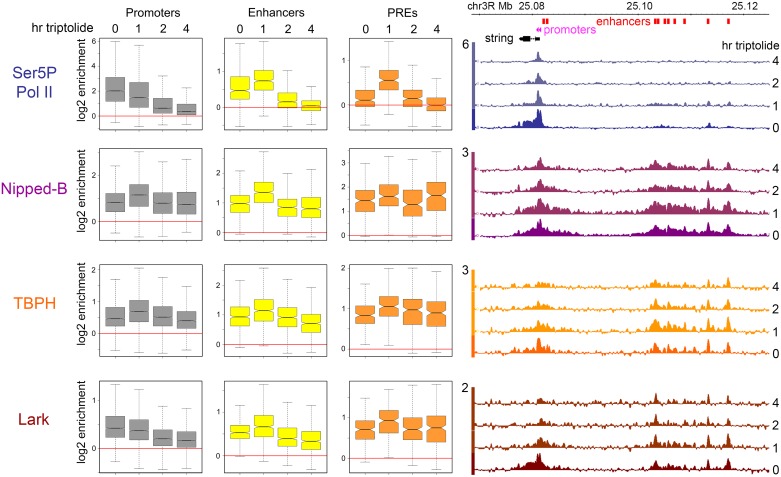
Inhibition of transcription initiation with triptolide does not ablate binding of Nipped-B, TBPH and Lark to gene regulatory sequences in BG3 cells. The boxplots show the average ChIP-seq enrichment of Ser5P Pol II, Nipped-B, TBPH and Lark at active promoters, extragenic enhancers, and PREs in control cells, and cells treated with 10 μM triptolide for 1, 2, and 4 hours. The genome browser tracks at the right show the log2 ChIP-seq enrichment for the same proteins at the *string* gene and enhancers over the same time course of triptolide treatment.

**Fig 6:**
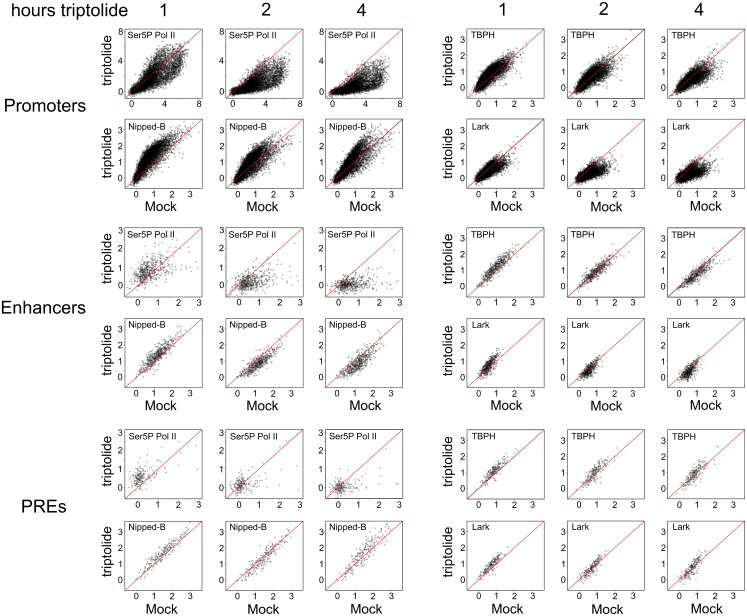
Effects of triptolide treatment on Ser5P Pol II, Nipped-B, TBPH and Lark occupancy at active promoters, extragenic enhancers and PREs in BG3 cells. The log2 ChIP-seq enrichment for Ser5P Pol II, Nipped-B, TBPH and Lark at all individual active promoters, enhancers and PREs in control untreated (Mock) cells is plotted against the enrichment after treatment of cells with 10 μM triptolide for 1, 2 and 4 hours.

The triptolide experiment results are shown in Figs 5 and 6. Ser5P Pol II shows the expected decreases at active promoters over time, although a small amount remains at some promoters even after 4 hours, as illustrated by the boxplots in Fig 5 and dot plots in Fig 6. We presume that the remainder is stably paused Pol II that only slowly enters into elongation. Consistent with this idea, there is still a small amount of Ser5P Pol II in the body of the *string* gene (Fig 5) and other highly expressed genes even after 1 hour of triptolide treatment, which we posit is stably paused Pol II [30] that has entered into elongation after blocking new initiation. There are significant differences between promoters in the disappearance of Ser5P Pol II from promoters, as illustrated by the more rapid decrease at the distal compared to the proximal *string* promoter (Fig 5 and S9 Fig). Taken together, these results confirm that triptolide treatment inhibits transcription as expected.

Unexpectedly, there is a transitory increase in Ser5P Pol II at enhancers and PREs after 1 hour of triptolide treatment that disappears after 2 hours, and decreasing to little or no binding by 4 hours (Figs 5 and 6). We theorize that triptolide treatment releases some Ser5P Pol II from promoters and/or gene bodies, which is then recruited to enhancers and promoters by protein interactions. It is unlikely that Pol II initiates transcription at enhancers and PREs in the presence of triptolide.

Nipped-B shows a substantially different response to triptolide treatment than Ser5P Pol II. Instead of a drop at the promoter, there is a transient increase after 1 hour, with a return close to the starting level by 2 hours (Figs 5 and 6). After 4 hours, some promoters still show an increase, while others show a decrease (Fig 6) with only a slight decrease in the median level (Fig 5). S8 Fig quantifies the changes in Nipped-B and Ser5P Pol II enrichment caused by triptolide treatment at the two *string* promoters, which show an 80 to 90% decrease in Ser5P Pol II enrichment after 2 hours, and 30% decrease in Nipped-B.

There is a similar overall pattern for Nipped-B binding in response to triptolide treatment at enhancers and PREs, with modest increases after 1 hour, and small decreases or little change in enrichment after 2 hours and 4 hours (Figs 5 and 6). Thus triptolide treatment largely halts transcription, but does not ablate Nipped-B binding to the gene regulatory sequences even after 4 hours of treatment.

TBPH shows a similar response to triptolide treatment as Nipped-B, with transitory small increases at promoters, enhancers and PREs, and return to levels close the starting levels by 2 hours (Figs 5 and 6). There is a more pronounced decrease at promoters and enhancers by 4 hours, although the remaining levels are only slightly lower than at the start. The *string* locus approximates the general pattern, as shown by the ChIP-seq browser tracks in Fig 5, except there are not increases at either promoter after 1 hour (S9 Fig). TBPH enrichment is reduced ~2-fold at the *string* promoters after 4 hours of triptolide treatment (S9 Fig) which is a larger decrease than average (Figs 5 and 6). The stable retention of TBPH at the regulatory sequences after 4 hours of blocking transcription indicates that protein or DNA interactions predominate over RNA binding in maintaining TBPH occupancy.

Unlike Nipped-B and TBPH, Lark responds similarly to Ser5P Pol II to triptolide treatment at promoters, showing a steady decrease over time (Figs 5 and 6). This is consistent with the idea that nascent RNA at promoters contributes significantly to Lark binding. However, the Lark response to triptolide treatment is similar to TBPH at enhancers and PREs, with a transitory increase at 1 hour, and modest reductions after 4 hours of treatment (Figs 5 and 6).

The above findings indicate that with the exception of Lark at promoters, continued transcription is not required to maintain Nipped-B, TBPH and Lark binding at gene regulatory sequences. This appears to contradict the idea that recognition of nascent RNA by TBPH governs recruitment of Nipped-B and cohesin. However, it is still possible that binding of TBPH to the first nascent transcripts at a gene when the gene is activated recruits the sister chromatid cohesion proteins, and that this recruitment leads to stable complexes that do not require continued transcription to maintain binding.

### TBPH and Lark interact specifically with nascent RNAs from cohesin-binding genes in vitro

The findings that (1) TBPH and Lark bind to regulatory sequences that are transcribed at low levels, (2) that blocking transcription with triptolide does not ablate TBPH and Lark binding to regulatory sequences, and (3) that depletion of Nipped-B reduces TBPH and Lark binding suggests that interactions of the RNA-binding proteins with other proteins predominate over their interactions with RNA in maintaining their binding to chromosomes. Nevertheless, the findings that TBPH depletion reduces Nipped-B association with genes, enhancers, and PREs, and that UG repeats are highly enriched in the promoter-proximal nascent RNAs of Nipped-B and cohesin binding genes are consistent with the idea that TBPH binding to nascent RNAs may be important for initiating Nipped-B and cohesin binding at these genes. Even though Lark depletion does not cause an overall reduction in Nipped-B and cohesin binding, transcription facilitates Lark association with promoters. Together these finding suggest that both RNA-protein and protein-protein interactions participate in determining association of the RNA-binding proteins, Nipped-B and cohesin with genes and regulatory sequences.

**Fig 7:**
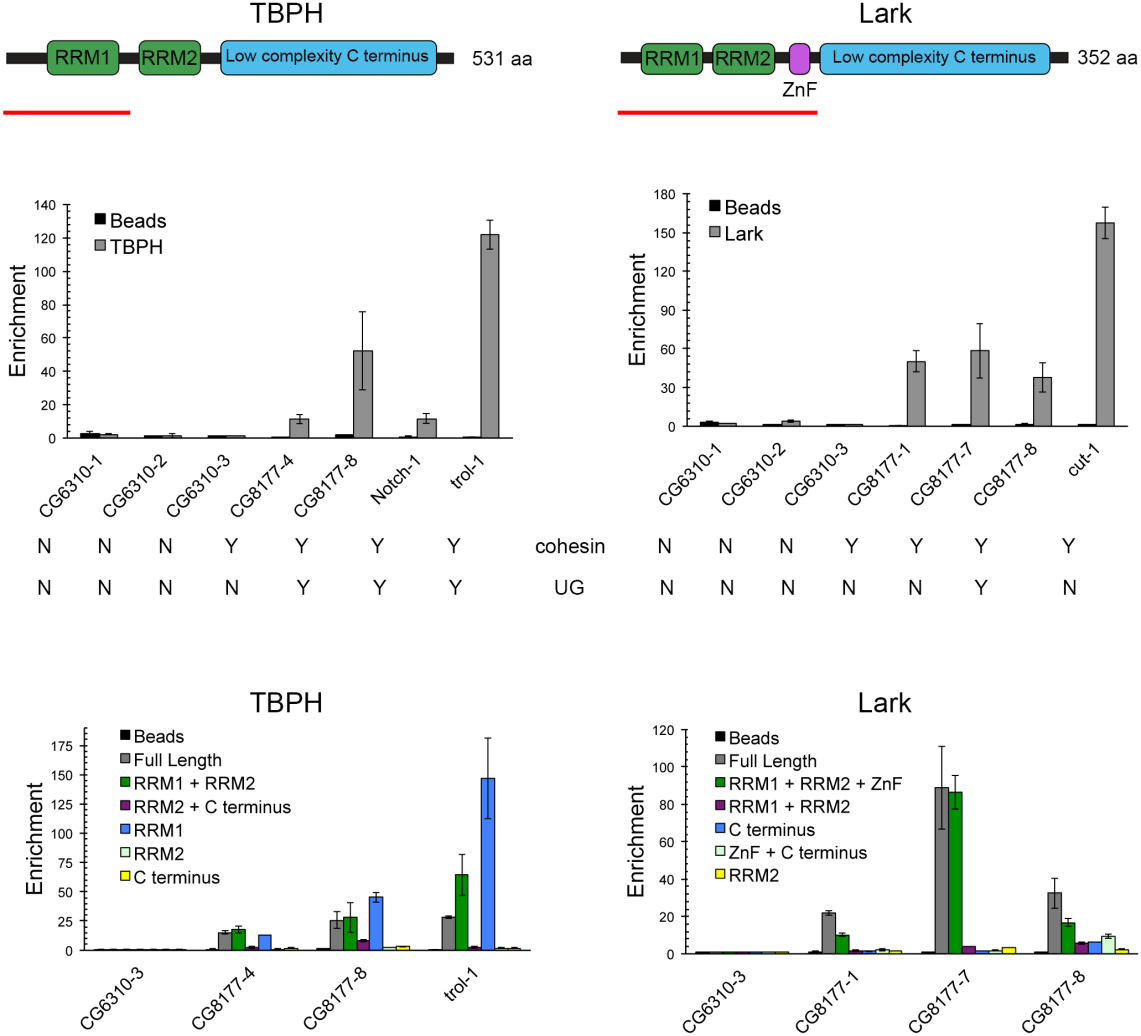
TBPH and Lark preferentially bind RNAs from cohesin-binding genes in vitro using known RNA-binding domains. Diagrams of the TBPH and Lark protein structures with known sequence domains shown at the top: RRM, RNA Recognition Motif; ZnF, Zinc Finger. The smallest fragments tested that bind RNA in vitro are underlined in red. The bar graphs below the protein diagrams show examples of RNA-binding competition experiments with TBPH (left) and Lark (right). Whether or not the RNA is from a cohesin-binding gene or contains UG repeats is indicated (Y = yes, N = no). The sequences of all short RNAs tested from cohesin-binding and non-binding genes are in S1 Table, along with a summary of their abilities to bind TBPH and Lark derived from multiple experiments. As detailed in the text, soluble His_6_-SUMO fusion proteins were immobilized on NTA-Zn^2+^ agarose beads and incubated with equimolar mixtures of the indicated short RNAs. RNAs that were retained after washing were quantified by real-time PCR and binding was defined by enrichment relative to the amount of the CG6310-3 control RNA, which does not bind to either protein. All RNA fragments were tested in two independent binding experiments with freshly made fusion proteins, and each RNA was measured twice in each experiment. Error bars are the standard deviations of all measurements in all experiments. Enrichment of 10-fold or greater is defined as specific binding when recorded in S1 Table. The bottom bar graphs show example RNA-binding experiments with the indicated fragments of TBPH and Lark to map the domains that bind RNA. SDS-PAGE characterization of the immobilized protein fragments and the residues contained within each fragment are shown in S10 Fig. For truncated proteins, RNA enrichment greater than or equal to half the enrichment obtained with the full-length protein in the same experiment was defined as binding. With TBPH, the RRM1-containing region is necessary and sufficient for RNA-binding. For Lark, the zinc finger (ZnF) is required in addition to the RRM-containing domain. It is unknown if one or both of the Lark RRM domains is required to bind RNA.

We conducted in vitro RNA binding assays with TBPH and Lark to test the ideas that they interact directly and specifically with RNAs produced by genes that bind Nipped-B and cohesin. TBPH and Lark have similar structures, with two RRM (RNA recognition motif) domains near the N terminus and low-complexity sequences in the C terminal half (Fig 7). Lark also has a CCHC zinc finger between RRM2 and the low complexity region (Fig 7). The full-length proteins and multiple truncated forms were expressed as soluble His_6_-SUMO-fusion proteins in *E*. *coli* and bound to NTA-Zn^2+^ beads (S10 Fig).

To test if TBPH and Lark specifically bind RNAs produced by cohesin-binding genes we performed in vitro binding reactions using a competition strategy [31] in which a mixture of several short RNAs ~50 nt in length from genes that bind cohesin (*CG8177*, *cut*, *Notch*, *trol*), and from two genes that do not (*CG6310*, *CG13089*) were incubated with the proteins bound to beads (S1 Table). After washing and elution, the retained RNAs were quantified by qRT-PCR as shown by the examples in Fig 7.

TBPH beads retained five of the 20 RNAs that were tested (S1 Table, Fig 7). Four of the bound RNAs contained UG repeats, and one did not, while all 15 RNAs that did not bind TBPH, including all seven from the *CG6310* and *CG13089* negative controls, lacked UG repeats (S1 Table, Fig 7). By Fisher’s exact test, the probability of the preference for UG-repeat containing RNAs occurring by chance is 0.001, consistent with prior reports that TBPH recognizes UG repeats [20].

Lark showed a different RNA-binding pattern than TBPH. Of the 20 RNAs tested for TBPH binding, Lark bound only two of the five bound by TBPH, and three that did not bind TBPH (S1 Table, Fig 7). Short sequence homology searches revealed that the five RNAs retained by Lark contain one or more copies of a CGUUC pentanucleotide sequence. To determine if this contributes to Lark binding, we tested ten short RNAs from the *rho* and *path* cohesin-binding genes, half containing CGUUC and half containing UG repeats (S1 Table). Combining the data, we find no correlation between binding to Lark and the CGUUC sequence (p = 0.46, Fisher’s exact test). There is, however, a correlation with UG repeats and Lark binding (p = 0.03). Thus UG repeats may contribute to Lark binding, but are neither sufficient nor required. Lark did not bind any of the RNAs from genes that do not bind cohesin and 10 of the 23 RNAs from cohesin-binding genes. MEME pattern analysis [32] of the 10 Lark-binding and 20 non-binding RNAs did not reveal enrichment of any other sequence motifs at p < 0.05, suggesting that secondary structure may be critical for Lark binding. Although these binding experiments did not identify a clear consensus sequence for Lark binding, prior studies suggest that Lark binding regions are A-rich [27] and that CU dinucleotides promote binding of the RBM4 orthologs of Lark to splice junctions [33, 34].

Experiments with the truncated forms of TBPH confirm that TBPH binds RNAs from cohesin-binding genes through the N terminal RRM1 domain, similar to the mammalian TDP-43 homolog [35] (Fig 7). We conducted in vitro RNA binding experiments with truncated TBPH proteins (S10 Fig) and mixtures of RNAs that do and do not bind the full-length proteins. All TBPH fragments containing RRM1 selectively bind the RNAs with UG repeats, but none of the truncated proteins lacking RRM1 bind any RNAs (Fig 7).

With Lark, all truncated forms that contain the zinc finger bind RNAs, while those lacking the zinc finger do not (Fig 7). Thus the zinc finger is required to bind RNA. A fragment containing the zinc finger and lacking both RRM motifs also does not bind RNAs. This suggest that at least one RRM domain is required in addition to the zinc finger for binding. It may be that both an RRM domain and the zinc finger contact RNA, but it is also possible that the C terminal domain inhibits RNA binding by the zinc finger in the absence of RRM domains.

### TBPH and Lark do not bind enhancer DNA

TBPH can bind TG repeats in single-stranded DNA (ssDNA) but not in double-stranded DNA (dsDNA) in vitro [20]. We examined the possibility that TBPH and Lark might recognize regions of single- or double-stranded DNA in enhancers and PREs using published genome-wide potassium permanganate (KMnO_4_) footprinting data for BG3 cells [36] that detects single-stranded DNA (S11 Fig). With the exception of a weak correlation (0.38) between the levels of Lark and single-stranded DNA at PREs, this analysis indicates that it is unlikely that TBPH and Lark bind ssDNA at poorly transcribed regulatory sequences. A strong correlation between Ser5P Pol II ChIP-seq enrichment and ssDNA levels at promoters (0.79) provides a positive control for this analysis.

We used the TBPH and Lark protein beads in competitive binding assays, similar to the RNA-binding experiments, with several double-stranded DNA fragments from the *cut* and *Wrinkled* gene super-enhancer regions to look for binding of the RNA-binding proteins to dsDNA (S11 Fig). Despite including fragments at TBPH and Lark ChIP-seq peaks we were unable to detect significant binding of the dsDNA fragments.

### Nipped-B protein interacts with TBPH and Lark

**Fig 8:**
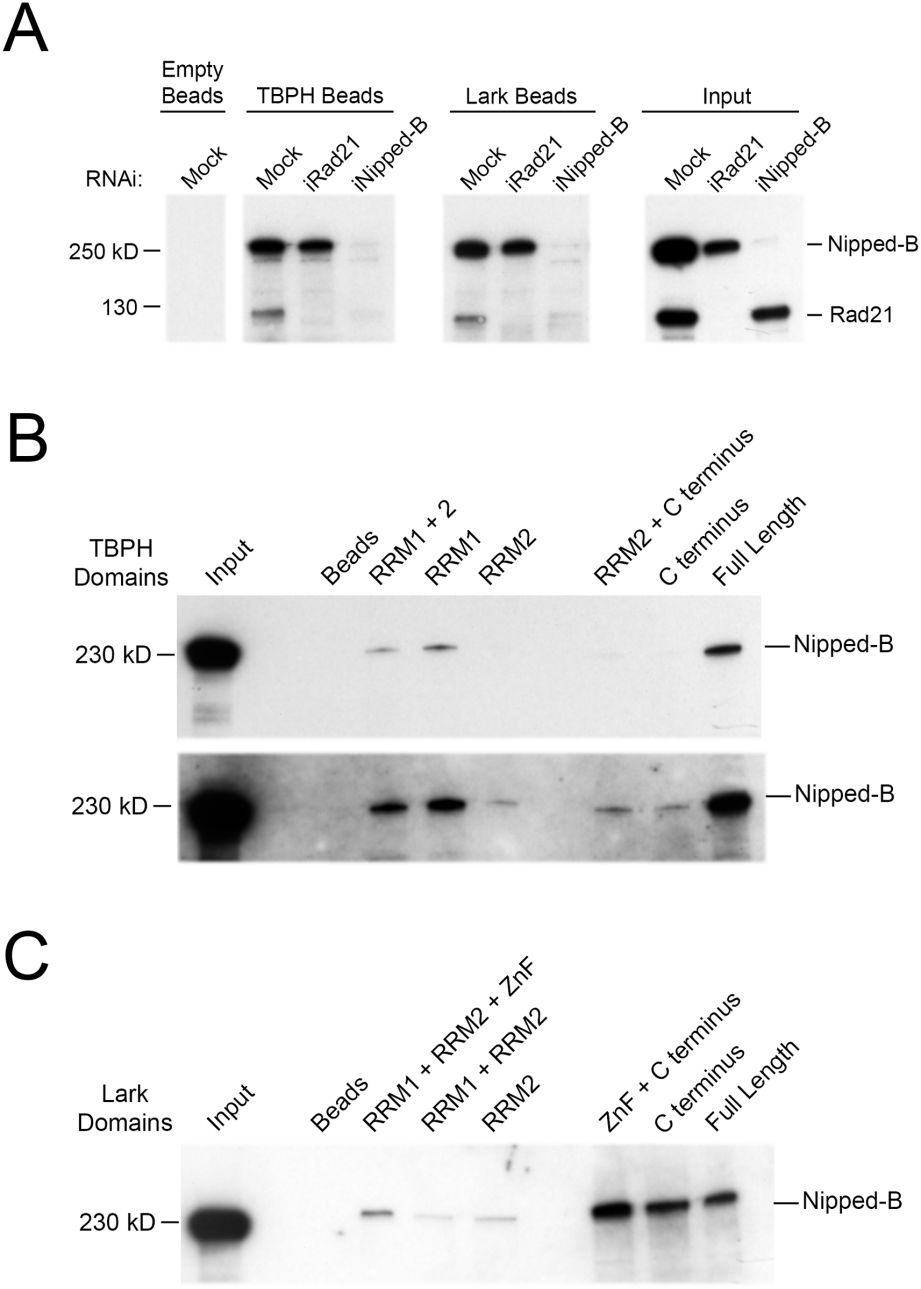
TBPH and Lark interact with Nipped-B. (**A**) The western blots show the binding of Nipped-B and cohesin (Rad21) in BG3 cell nuclear extracts to NTA-Zn^2+^ agarose beads with immobilized His_6_-SUMO-TBPH and -Lark fusion proteins probed with both Nipped-B and Rad21 antibodies. The western on the far right (Input) shows the Nipped-B and Rad21 proteins in the BG3 cell nuclear extracts used for the binding experiments. Nuclear extracts were prepared from control (Mock) cells and cells depleted for Rad21 and Nipped-B by RNAi. The results shown for mock nuclear extract are representative of five independent experiments. The results shown for nuclear extracts depleted for Rad21 and Nipped-B are representative of two technical replicates. S12 Fig shows that native TBPH and Lark co-immunoprecipitate with Nipped-B from nuclear extracts, that Lark and TBPH do not co-precipitate, and that pre-treatment of BG3 nuclear extract with ribonucleases does not prevent binding of Nipped-B to TBPH and Lark beads. (**B**) Western blot of Nipped-B binding to the indicated TBPH fragments and full-length TBPH to map the protein domains interacting with Nipped-B. The bottom panel is a longer exposure of the same blot to show low levels of Nipped-B binding to some fragments. The fragments are shown in S10 Fig. Most of the Nipped-B binding occurs with the RRM1 domain of TBPH. The blots shown are representative of three independent experiments. (**C**) Western blot of Nipped-B binding to the indicated fragments of Lark and full-length Lark. The fragments are shown in S10 Fig. The blot shown is representative of three independent experiments.

In vitro binding experiments indicate that TBPH and Lark both interact specifically with Nipped-B protein. The protein beads with TBPH and Lark were incubated with BG3 cell nuclear extracts, and Nipped-B and cohesin (Rad21) in the extracts retained by the beads after extensive washing were detected by western blotting (Fig 8A). The binding reactions contained 190 mM salt, 0.83% Triton-X-100 detergent and imidazole to minimize non-specific interactions. Under these conditions, Lark and TBPH beads both retain Nipped-B and cohesin, while empty beads do not (Fig 8A).

Immunoprecipitation of Nipped-B from nuclear extracts under similarly stringent conditions co-precipitates TBPH, and Lark immunoprecipitation co-precipitates Nipped-B (S12 Fig). This confirms that native TBPH and Lark proteins also interact with Nipped-B when TBPH and Lark are present at substantially lower concentrations than in the affinity chromatography experiments. Addition of ethidium bromide to the nuclear extract did not reduce co-precipitation of TBPH with Nipped-B antiserum indicating that the interaction does not require DNA (S12 Fig). Interaction of Nipped-B and cohesin with TBPH and Lark also does not involve interactions between TBPH and Lark because TBPH and Lark do not co-precipitate with each other from nuclear extracts (S12 Fig). Interactions between the RNA-binding proteins and Nipped-B do not require RNA because pre-digestion of nuclear extracts with RNase A and RNase T1 did not alter the binding of Nipped-B to TBPH and Lark beads (S12 Fig).

We used nuclear extracts of BG3 cells depleted for Nipped-B and Rad21 to test if cohesin interacts with the RNA binding proteins through Nipped-B, or if Nipped-B interacts via cohesin. Neither RNA-binding protein binds cohesin (Rad21) when Nipped-B is depleted, but Nipped-B is retained in the absence of Rad21 (Fig 8A). Thus TBPH and Lark interact specifically with Nipped-B, and not with cohesin, which binds indirectly via interaction with Nipped-B. Although the simplest interpretation of these findings is that Nipped-B interacts directly with both RNA-binding proteins we cannot rule out the possibility that Nipped-B interacts indirectly through another protein present in the nuclear extracts.

We used truncated proteins (S10 Fig) to map the regions of TBPH and Lark that interact with Nipped-B. All TBPH fragments containing RRM1 interact with Nipped-B, and forms lacking RRM1 retain very low levels (Fig 8B). The Lark C terminal region with low complexity sequence binds Nipped-B, while only weak interactions occur with regions containing the RRM domains and the zinc finger (Fig 8C). The amounts of beads added to the binding reactions were adjusted so that the total protein mass was the same in all reactions. All protein fragments are soluble, suggesting that the proteins are likely to be properly folded. As shown above, fragments containing the RRM and zinc finger domains bind RNA, indicating that they are functional. The low-complexity C terminal domains are not predicted to have regular structures, but do not aggregate under the experimental conditions. These experiments show that the region of TBPH containing RRM1 and the C terminal low complexity domain of Lark interact with Nipped-B, providing further evidence for the specificity of their interactions with Nipped-B. The interactions between both RNA-binding proteins and Nipped-B suggest mechanisms for the recruitment of Nipped-B by TBPH at active genes and regulatory sequences, and for how Nipped-B can facilitate binding of TBPH and Lark to promoters and poorly-transcribed regulatory sequences. The lack of interactions between the RNA-binding proteins and cohesin indicates that the effects of the RNA-binding proteins on cohesin levels at genes and regulatory sequences likely reflect changes in Nipped-B occupancy.

## Discussion

The experiments described above were motivated by the prior finding that genes that bind Nipped-B and cohesin, in contrast to genes that bind little or no cohesin, produce nascent RNAs that contain UG repeats proximal to the transcription start site [19]. This gave rise to the hypothesis that RNA-binding proteins recognizing these nascent RNAs contribute to the recruitment of Nipped-B and cohesin to a subset of active genes. Here we tested this idea by mapping the binding of the TBPH and Lark RNA-binding proteins genome-wide by ChIP-seq, measuring how depletion of these RNA-binding proteins affect Nipped-B and cohesin binding to genes, and testing their ability to interact with specific RNAs and Nipped-B and cohesin in vitro.

**Fig 9:**
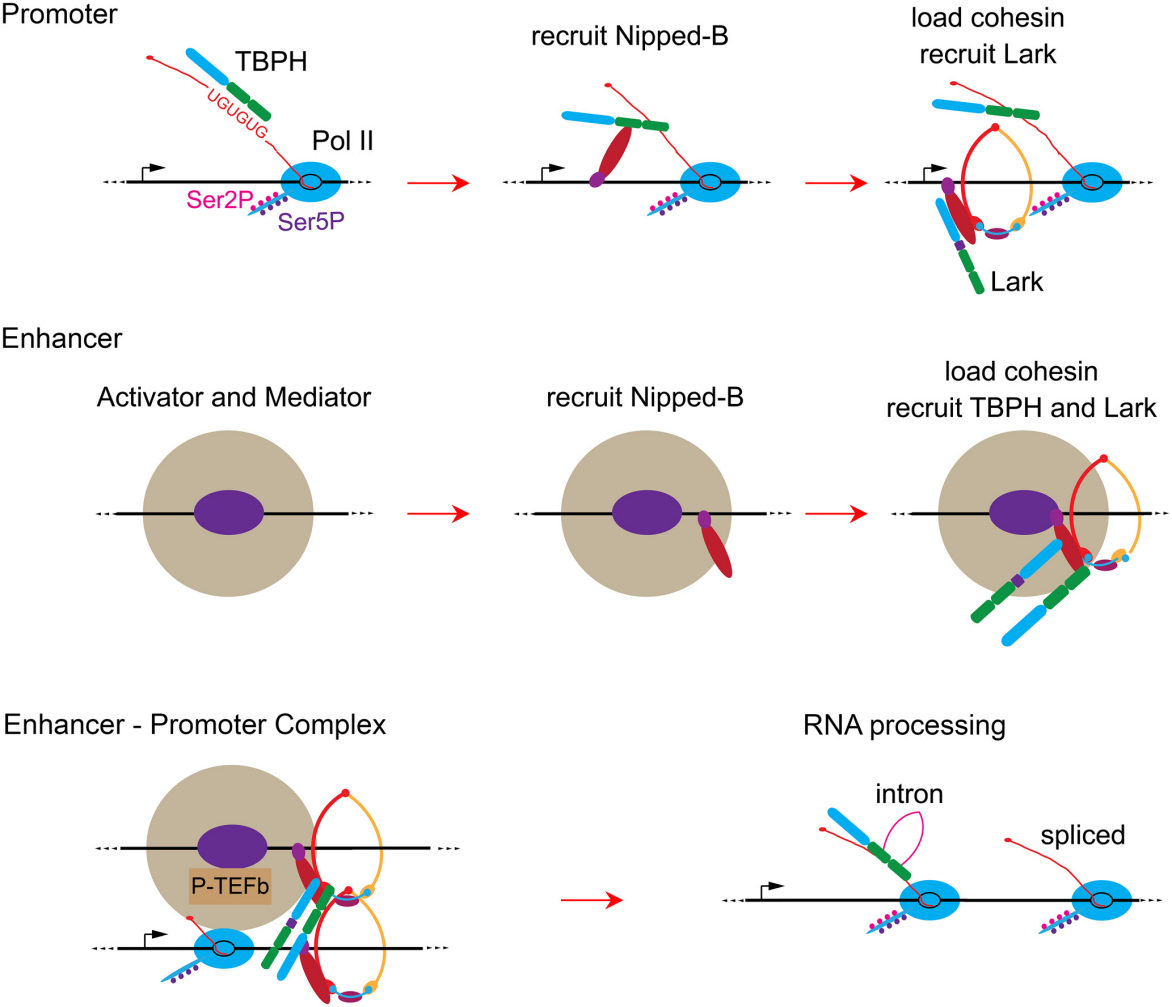
Hypothetical models for roles of TBPH and Lark in Nipped-B and cohesin binding to genes and enhancers, enhancer-promoter interactions, and processing of nascent RNA transcripts. At promoters (top row) we posit that TBPH binds to UG repeats in the first nascent transcripts produced by elongating Pol II (Ser2P / Ser5P) when a gene is initially activated. TBPH then recruits Nipped-B, which interacts with DNA, loads cohesin and recruits the Lark RNA-binding protein. At enhancers (middle row) activator proteins (purple oval) recruit Mediator (large tan circle) and Nipped-B. Nipped-B then loads cohesin and recruits TBPH and Lark, and TBPH stabilizes binding of the complex. The protein complexes at the enhancers and promoters form enhancer-promoter complexes (bottom row) that are stable for hours even in the absence of new transcription initiation. TBPH contributes to their stability, while Lark destabilizes cohesin and Nipped-B binding, particularly to the promoter. P-TEFb in the enhancer-promoter complex phosphorylates paused Pol II and the pausing factors (not depicted), leading to transcriptional elongation (lower right). Some TBPH and Lark present in the enhancer-promoter complex binds to the nascent RNA produced by the elongating polymerase and can facilitate RNA processing. For example, they can regulate intron removal by splicing as depicted in the lower right diagram, in addition to other processes.

Our current thinking based upon the results is illustrated in Fig 9. We hypothesize that at promoters, TBPH binds UG repeats in initial nascent RNA transcripts (pioneer transcripts) when a gene is first activated, and then recruits Nipped-B, which in turn loads cohesin and aids binding of Lark (Fig 9). These initial steps lead to stable binding of Nipped-B, TBPH and Lark to promoters. At enhancers and PREs, where there is little transcription, the binding order is likely different. We posit that activator and silencing proteins first recruit Nipped-B, which then loads cohesin and aids binding of both RNA-binding proteins (Fig 9). Interactions between enhancer and promoter-bound protein complexes then facilitate enhancer-promoter looping.

We thus posit that Nipped-B and cohesin are recruited independently to enhancers and promoters, and stable enhancer-promoter interactions are established when they are present at both (Fig 9). In this view, basal transcription from a promoter can continuously “test the waters” to see if there is an available enhancer with which to interact and establish an activated level of transcription. Once established, the interactions of TBPH and Lark with Nipped-B and other proteins at enhancers and promoters maintain their association with chromosomes even in the absence of continued transcription. This transcription-independent binding could serve to reduce fluctuations in the levels of transcription over time.

In addition to the original finding that cohesin-binding genes produce nascent RNAs with promoter-proximal UG repeats [19], three lines of evidence presented here support the idea that TBPH binding to nascent RNAs recruits Nipped-B and cohesin to specific genes: (1) TBPH specifically selects RNAs with UG repeats from RNA mixtures in vitro, (2) TBPH binding to chromosomes correlates strongly with Nipped-B binding at promoters by ChIP-seq, and (3) TBPH interacts with Nipped-B in affinity chromatography and co-immunoprecipitation experiments.

On the face of it, the reduction in Nipped-B and cohesin binding genome-wide upon TBPH depletion is also consistent with the idea that TBPH binding to nascent RNA recruits Nipped-B to specific genes. However, two additional pieces of evidence indicate that this decrease in Nipped-B binding more likely reflects a role for TBPH in stabilizing Nipped-B binding after it has been established. First, TBPH binds to poorly-transcribed enhancers and PREs, and TBPH depletion reduces Nipped-B and cohesin at these regulatory elements. More importantly, inhibition of transcription for up to four hours with triptolide does not ablate TBPH or Nipped-B binding to genes and regulatory sequences. We thus posit that once established, TBPH and Nipped-B are included in stable protein complexes at promoters, enhancers and PREs that do not require RNA for their maintenance (Fig 9). The presence of these transcription-independent complexes interferes with our ability to provide definitive evidence for the idea that TBPH binding to nascent RNA initiates binding of Nipped-B to specific genes. Nevertheless, we still currently favor this idea based on the in vitro binding of TBPH to Nipped-B and UG-repeat containing RNAs. It should be possible to test if TBPH binds promoter before Nipped-B at genes with TG repeats that can be suddenly activated, although we currently do not know of genes amenable to these experiments.

Although depletion of Lark has complex bimodal effects on binding of Nipped-B, it is possible that it could also contribute to the original recruitment of Nipped-B to specific genes. Lark also specifically selects RNAs from cohesin-binding genes in vitro, co-localizes with Nipped-B and cohesin genome-wide, and interacts with Nipped-B in vitro. The difference in the effects of TBPH and Lark depletion on Nipped-B levels we observed likely reflect differences in their ability to stabilize established Nipped-B binding, not in their ability to initially recruit Nipped-B.

An alternative explanation for the selective binding of TBPH and Lark to RNAs from Nipped-B and cohesin-binding genes and the enrichment of UG repeats in the nascent transcripts is that these genes have evolved to produce RNA sequences that aid recruitment of TBPH and Lark to facilitate RNA processing. An unexpected finding from a prior study is that genes that bind higher levels of cohesin produce an average of 2-fold more steady state mRNA molecules per actively transcribing Pol II complex than genes that bind little or no cohesin [17]. Thus whether or not TBPH and Lark initially recruit Nipped-B to a gene, TBPH and Lark may increase the efficiency of RNA processing specifically at cohesin-binding genes (Fig 9). The TDP-43 ortholog of TBPH is involved in multiple types of RNA processing [37, 38]. Lark is critical for appropriate subcellular localization of some RNAs in oogenesis [39] and the RBM4 homologues of Lark influence 5’ splice site usage and exon selection [34].

The observed association of TBPH and Lark with enhancers and PREs was unexpected because they produce low levels of nascent RNA as measured by PRO-seq. Binding of TBPH and Lark to enhancers and PREs is also surprisingly stable for hours after blocking transcription, indicating that even nascent RNA at promoters, which can contact enhancers through looping interactions, is not required for their binding at enhancers. Although we posit that Nipped-B recruits TBPH and Lark to enhancers and PREs, strong Nipped-B depletion only partially reduces their binding, indicating that other factors may help maintain their binding. Our experiments suggest that TBPH and Lark are unlikely to bind single- or double-stranded DNA, and we thus posit that they also interact with other proteins such as transcriptional activator and repressor complexes. Experiments in primary mouse liver cells argue that cohesin stabilizes large protein-DNA complexes at enhancers [40] and the results here indicate that these complexes likely include RNA-binding proteins.

It remains to be resolved how Nipped-B and cohesin are initially recruited to enhancers and PREs. Although their initial recruitment to active promoters could lead to their transfer to enhancers via enhancer-promoter looping, their presence at PREs, which silence adjacent genes, indicates that active promoters are not essential for binding. We thus favor the idea that transcriptional activator and repressor complexes facilitate binding of Nipped-B and cohesin to enhancers and PREs (Fig 9). In this view, recruitment of Nipped-B and cohesin to a promoter by RNA-binding proteins associated with nascent RNA could then make that promoter a preferred enhancer target through interactions between Nipped-B and cohesin. In the absence of an available enhancer with Nipped-B and cohesin, transcription would remain at a basal level.

Binding of TBPH and Lark to both promoters and enhancers raises the possibility that they might also facilitate enhancer-promoter looping (Fig 9). The low complexity C terminal domains of these proteins interact with themselves and other proteins, and often form aggregates under disease conditions [41]. Such interactions could thus help stabilize long-range enhancer-promoter loops. This idea, however, will be difficult to test because TBPH depletion reduces the levels of Nipped-B and cohesin, which facilitate looping [5]. Other RNA-binding proteins with similar structures might also be present and support looping in the absence of Lark or TBPH.

The high stability of Nipped-B, TBPH and Lark binding to promoters and enhancers in the absence of transcription is relevant to live imaging studies showing that enhancers control the frequency of transcriptional bursting in Drosophila embryos and mammalian cells [42, 43]. These findings indicate that transcription is not continuous, but occurs in bursts. The bursts have the same amplitudes, and thus the frequency with which bursts occur varies between enhancers and determines enhancer strength. Transcription-independent binding of Nipped-B, which facilitates gene activation by long-range enhancers [44] suggests that enhancer-promoter loops may be stable over extended periods. If this is the case, then the frequency of transcriptional bursting is not determined by the frequency of enhancer-promoter contact, but by the availability of other enhancer or promoter-specific factors that promote the transition of paused Pol II into elongation. Consistent with this idea, Nipped-B association with enhancers and promoters is similarly stable upon blocking transcription initiation, while the rate of reduction of paused Pol II at promoters varies.

Against this idea, forcing enhancer-promoter looping increases transcriptional burst frequency at the β-globin gene [42]. If this holds true at other genes, then the different burst frequencies observed with various enhancers in Drosophila embryos [43] likely reflect different frequencies of enhancer-promoter contact. If so, the stable transcription-independent presence of Nipped-B at both enhancers and promoters indicates that the presence of Nipped-B and cohesin at enhancers and promoters does not determine the frequency of enhancer-promoter looping. In this case, other factors, which may include the ability of different activators to recruit Mediator and P-TEFb complexes (Fig 9) determine the frequency and stability of looping interactions.

## Materials and Methods

### Affinity chromatography to identify proteins binding RNA leader sequences

Leader sequences (200 to 610 bp) from genes containing long TG repeats (*cut*, *CG8177*) and genes lacking TG repeats (*CG13089*, *CG6310*) were amplified from genomic DNA with AccuPrime Pfx enzyme (Life Technologies) and cloned into the pCR-Blunt II-TOPO vector (Life Technologies) and the sequences confirmed by sequencing (Retrogen Inc). Clones were linearized by digestion with *Bam*HI, and used as templates for *in vitro* transcription using the mMESSAGE mMACHINE T7 Kit from Ambion, which generates capped RNA. RNAs were labeled at the 3’ end with biotin using the Pierce RNA 3’ End Biotinylation Kit. The biotinylated RNAs were bound to streptavidin magnetic beads (Invitrogen Dynabeads MyOne Streptavidin C1) according to the manufacturer’s instructions.

To prepare nuclear extract, BG3 cells were grown to a density of approximately 5 x 10^6^ per mL and collected by scraping and centrifugation at 300g for 5 min at 4°. The cell pellet was washed with 15 mL phosphate buffered saline (PBS) by suspension and centrifugation, and then with 15 ml of hypotonic buffer [10 mM Hepes KOH pH 7.9, 50 mM KCl, 1.5 mM MgCl_2_, 0.5 mM dithiothreitol (DTT), 0.5 mM phenylmethylsulfonyl fluoride (PMSF)]. Cells were suspended in three times the cell pellet volume of hypotonic buffer and incubated on ice 5 min prior to homogenization with 60 strokes in a ground glass homogenizer. Nuclei were collected by centrifugation at 2,000g for 5 min at 4°. The nuclear pellet was washed with an equal volume of hypotonic, and then suspended in two volumes of extraction buffer [10 mM Hepes, pH 7.9, 0.4 M NaCl, 1.5 mM MgCl_2_, 5% glycerol (v/v), 0.5 mM DTT, 0.5 mM PMSF]. The NaCl concentration was adjusted to 0.35 M, and nuclei were incubated for 30 min on ice. Insoluble material was removed by centrifugation at 16,000g for 60 min at 4°. Nuclear extract was adjusted to 20% glycerol (v/v) and rapidly frozen on dry ice and stored at -80°.

To identify proteins that bind RNA leader sequences, 1.2 mg of nuclear extract was incubated with 80 pmol of RNA leader bound to 50 microliters of magnetic beads in binding buffer [10 mM Hepes KOH pH 7.9, 1 mM MgCl_2_, 0.8 mM magnesium acetate, 10% (v/v) glycerol, 1 mM dithiothreitol (DTT), 0.1 mg per mL tRNA, 0.5 mg per mL heparin, 75 mM NaCl, cOmplete protease inhibitor cocktail (Roche), RNasin (Promega)] in a final volume of 0.8 mL at 30°C for 40 min. After binding, the beads were washed with RNA wash buffer [10 mM Hepes KOH pH 7.5, 1 mM MgCl_2_, 0.8 mM magnesium acetate, 10% (v/v) glycerol, 1 mM DTT] 4 times at room temperature. Washed beads were boiled in SDS-PAGE loading buffer and the proteins were separated by SDS-PAGE and visualized by silver staining (Thermo Scientific Pierce Silver Stain for Mass Spectrometry). Protein bands present only in the lanes using leaders from the cohesin-binding genes (*cut*, *CG8177*) and the equivalent region in the lanes from the leaders from genes that don’t bind cohesin (*CG13089*, *CG6310*) were excised from the gel for identification by mass spectrometry in the Proteomics and Microchemistry Core Facility at Memorial Sloan-Kettering Center. Lark was identified with a 99% probability in the proteins binding *CG8177* and was not detected in control leader lanes.

### TBPH and Lark antibodies

TBPH protein bound to NTA-Zn^2+^ agarose beads (see below) was washed with Urea Wash Buffer [8 M urea, 10 mM Tris base, 100 mM NaH_2_PO_4_] and eluted with stripping buffer [8 M Urea, 10 mM Tris base, 100 mM NaH_2_PO_4_, 100 mM EDTA, 0.1% NP-40, 5% (v/v) glycerol, 300 mM KCl adjusted to pH 4.5]. Eluted protein was dialyzed in PBS containing 0.01% (v/v) Tween, 100 micromolar ZnCl_2_, and 0.5 mM PMSF and was used to immunize a guinea pig (Pocono Rabbit Farm and Laboratory, Canadensis, PA). Anti-Lark antibodies were prepared by expressing amino acids 3 to 235 of the Lark protein as a His_6_ fusion in bacteria using the pET-16b vector and immunizing a rabbit with purified protein.

### ChIP-seq

Multiple independent biological replicates were performed for all ChIP-seq experiments, except the triptolide time course, in which two technical replicates were performed for Lark ChIP-seq. The Nipped-B, SA and Rad21 antibodies were previously described [23]. RNAi western validation of the Lark and TBPH antibodies is shown in S1 Fig. The Ser5P Pol II rabbit monoclonal antibody was purchased from Cell Signaling Technology (#13523) and validated by western blots of cell extracts with and without triptolide treatments. BG3 cells were cultured and treated with double-stranded RNA for protein depletion by RNAi for four to five days as previously described [16]. The double-stranded RNAs to deplete Nipped-B and Rad21 are described elsewhere [16] and the oligonucleotide primers used to prepare templates for Lark and TBPH double-stranded RNA are shown in S1 Fig. Depletions were confirmed by western blots of whole cell protein extracts made in lysis buffer [8 M urea, 1% (v/v) NP-40, 40 mM Tris HCl pH 7.4]. Chromatin was prepared and immunoprecipitation was performed as described elsewhere [23, 24]. Chromatin was prepared from wing discs dissected from male *y w* crawling 3^rd^ instar larvae as described previously [18].

Ion Torrent sequencing libraries were prepared from input chromatin and ChIP samples as described in detail elsewhere [24]. Input and ChIP libraries were sequenced to generate ~10-fold genome coverage for each sample. The method and computer scripts for calculating enrichment of sequences in the ChIP samples relative to input are described and validated elsewhere [24] by comparing to Nipped-B and cohesin genomic ChIP using tiling microarrays [17, 18, 23] processed using either the TiMAT [45] or MAT [46] programs. Sequencing the ChIP and input libraries to at least 10-fold genome coverage is critical to obtain results that closely match those obtained using ChIP-chip in terms of both sensitivity and locations of peaks and broad domains [24]. The ChIP-seq data has been deposited in the GEO database (accession GSE83959).

The method used to quantify average ChIP-seq enrichment of active gene promoters and PREs is diagrammed in S4 Fig. The ave.chip.r script in R [47] used for this analysis is in S1 File, and the bed files for active promoters, enhancers, PREs, gene bodies, and random sites are in S2–S6 Files. Published PRO-seq (GEO accession GSE42397) and KMnO_4_-seq (GEO accession GSE46620) were analyzed using the sum.read.r script in S1 File. The Integrated Genome Browser (IGB) [48] and R were used in further data analysis and preparation of figures for ChIP-seq data.

### Expression of Lark and TBPH fusion proteins

Lark and TBPH full length and truncated proteins were cloned into the pCR-Blunt II-TOPO vector (Life Technologies). Inserts were released by digestion with *Xho*I and *Bam*H1 and sub-cloned into the pSMT3 vector [49] in Rosetta 2 *E*. *coli* cells (Novagen), and confirmed by DNA sequencing (Retrogen). Expression of the His_6_-SUMO fusion proteins was induced with 0.5 to 0.8 mM isopropyl β-D-1-thiogalactopyranoside (IPTG) for 4 hours at 25° during the linear growth phase. Cells were pelleted and suspended in lysis buffer [50 mM Tris HCl pH 7.5, 0.5 M NaCl, 5% (v/v) glycerol, 0.5 mM tris(2-carboxyethyl)phosphine (TCEP), 0.5 mM phenylmethylsulfonyl fluoride (PMSF), 0.1% (v/v) Triton X-100, 1 mg per mL lysozyme]. Cells were flash frozen in liquid nitrogen, thawed, and 10 U DNase I (Epicentre) added to the lysate. Lysate was incubated for 1 hr at room temperature and sonicated for up to 6 cycles of 30 seconds, and debris were removed by centrifugation at 16,000g for 15 min. The supernatant was incubated with NTA- Zn^2+^ agarose beads (Qiagen) for 1 hr at 4°, collected by gentle centrifugation and washed five times with wash buffer [50 mM Tris HCl pH 7.6, 0.5 M NaCl, 20 mM imidazole, 0.1% Triton X-100, 1 mM DTT, cOmplete protease inhibitor cocktail]. Beads were stored at 4° for up to two days in wash buffer before use. Examples of the expressed proteins are shown in S10 Fig.

### Lark and TBPH in vitro RNA and DNA binding experiments

Oligonucleotide primers were used in PCR reactions to amplify DNA templates from BG3 cell genomic DNA with a T7 promoter sequence at the 5’ end and an oligo B2 sequence (Affymetrix) at the 3’ end. RNAs (S1 Table) were synthesized by *in vitro* transcription of the templates using T7 RNA polymerase (Promega), and purified by chloroform extraction and ethanol precipitation. Equimolar RNA mixes (20 micrograms) were denatured by incubation at 65° for 3 min, and incubated with Lark or TBPH protein beads (1 microgram protein, S10 Fig) prepared as described above at 37° for 30 min at a total volume of 0.5 mL in RNA binding buffer [20 mM Hepes KOH pH 7.8, 100 mM KCl, 20 mM NaCl, 0.05% (v/v) NP-40, 10% (v/v) glycerol, 2 mM MgCl_2_, 2 mM DTT, RNasin, 0.1 mg per mL bovine serum albumin (BSA), 15 micrograms per mL heparin]. Beads were washed several times with binding buffer at room temperature, and the bound RNAs eluted by boiling in STE [1% SDS, 10 mM Tris HCl pH 7.5, 2 mM ethylenediaminetetraacetic acid (EDTA)]. Eluted RNA was subjected to chloroform extraction, collected by ethanol precipitation, and dissolved in 10 microliters H_2_O. cDNA was prepared from the RNA using SuperScript III Reverse Transcriptase using an oligo B2 primer and quantified by real-time PCR using RNA-specific primers as described elsewhere [16]. Binding to DNA fragments from *cut* and *Wrinkled* enhancers were performed similarly. S7 File is a bed file with the positions of the DNA fragments tested.

### Interactions of Nipped-B and cohesin with TBPH and Lark on beads

TBPH or Lark proteins (approximately 5 micrograms protein) bound to NTA-Zn^2+^ agarose beads (see above) were washed twice with Casein Blocking Solution [1% (w/v) casein, 2 mM DTT, cOmplete protease inhibitor cocktail in PBS] for 2 hours. Nuclear extracts (approximately 400 micrograms) prepared as described above were diluted 1:6 into Binding Buffer [1% Triton X-100, 2.5% BSA, 20 mM imidazole, 2 mM MgCl_2_, 2 mM DTT, cOmplete protease inhibitor cocktail, RNasin (Promega) in PBS] for a final volume of 0.85 mL and cleared of insoluble material by centrifugation at 16,000g for 10 min at 4°. The final concentrations in the diluted extract are: 1.67 mM Hepes KOH pH 7.9, 8.3 mM Na_2_HPO_4_, 1.5 mM KHPO_4_, 172 mM NaCl, 2.25 mM KCl, 1.95 mM MgCl_2_, 16.7 mM imidazole, 0.83% Triton X-100, 3.33% glycerol, and 2% BSA, giving an ionic strength greater than 190 mM, and a significant detergent concentration to decrease non-specific binding. Imidazole prevents non-specific binding of proteins to the NTA-Zn^2+^ beads. Cleared extracts were incubated with blocked TBPH or Lark beads for 2 hours 4°. Beads were washed twice with Binding Buffer (PBS with Triton X-100, imidazole and BSA), and twice with PBS only, before boiling in SDS-PAGE buffer and separating bound proteins by SDS-PAGE. Proteins were detected by Western blot. For ribonuclease treatment, nuclear extracts were treated with 0.2 mg per mL RNase A and 4 U per mL RNase T1 for 30 min at 37° prior to binding to TBPH or Lark beads.

### Co-immunoprecipitation experiments

Approximately 300 micrograms of nuclear extract (see above) was diluted 1:3 with Buffer 2 [50 mM Hepes KOH pH 7.9, 100 mM KCl, 2.5 mM MgCl_2_, 0.2% NP-40, 1 mM MnCl_2_, 1 mM DTT, 1 mM PMSF, cOmplete protease inhibitor cocktail]. The final concentrations in the diluted extract were: 36.7 mM Hepes KOH pH 7.9, 116.7 mM NaCl, 66.7 mM KCl, 2.5 mM MgCl_2_, 0.67 mM MnCl_2_, 0.133% NP-40, and 6.67% glycerol, giving a nominal ionic strength of 183.4 mM. The diluted extract was pre-cleared by incubation with Protein A agarose beads (Thermo Scientific) for 2 hours at 4°. Antibodies were added to pre-cleared nuclear extract and the reactions were incubated overnight at 4°. Immune complexes were collected by incubation with Protein A agarose beads for 4 hours at 4°, and the beads were washed five times with Buffer 2 before suspending in SDS-PAGE loading buffer, denaturing at 100° for 5 min and separating the proteins by SDS-PAGE. Precipitated proteins were detected by western blotting. For some experiments 50 micrograms per mL ethidium bromide was added to the nuclear extract prior to immunoprecipitation.

## Supporting Information

S1 Fig Western blots of BG3 cells depleted for TBPH, Lark and Nipped-B. BG3 cells were depleted for the indicated proteins for four to five days and 15 μg of total cell extracts were analyzed by western blotting. The blots shown are representative of at least three independent experiments. The panels on the left compares mock-depleted control cells (Mock) versus cells depleted for TBPH (iTBPH) using double-stranded RNA prepared using the primers in the table at the bottom. The blot was probed with antibodies against Nipped-B, Rad21, SA, TBPH (this study), Lark (this study) and actin antibodies. The panels in the middle compare control cells (Mock) to cells depleted for Lark (iLark) using dsRNA prepared using the primers shown in the table. The right panels compare control cells to cells depleted for Nipped-B (iNipped-B). Western blots with lanes loaded with 7.5 μg or 30 μg of extract gave equivalent results.
(TIF)

S2 Fig TBPH and Lark occupy Polycomb Response Elements (PREs) silencing the *vestigial* gene in BG3 cells. The genome browser view shows the *vestigial* gene (transcribed left to right) and the PREs (orange boxes) that silence it. The top ChIP-chip track (gray) shows H3K27me3 (histone H3 lysine 27 trimethyl) mark made by the PRC2 silencing complex in BG3 cells [25] with enrichment expressed as MAT [46] score. The brown Lark, orange TBPH and purple Nipped-B ChIP-seq tracks show the log2 enrichment for each protein, and are the average of two to three independent experiments. The bars underneath the ChIP-seq tracks indicate where enrichment is in the 95^th^ percentile for ≥150 bp.
(TIF)

S3 Fig Identification of Lark as a nuclear-localized RNA-binding protein that preferentially associates with RNAs from cohesin-binding genes. (**A**) Affinity chromatography strategy to identify RNA binding proteins in nuclear extracts that recognize a nascent RNA leader from a cohesin-binding gene. Nuclear extracts were prepared from cultured ML-DmBG3 (BG3) cells derived from larval central nervous system. The N terminal nascent RNA sequence from the *CG8177* gene contains a region with four UG repeats, and one with eight. *CG8177* is transcribed and binds Nipped-B and cohesin in both Sg4 (Schneider Line 2 derivative) and BG3 cells [23]. The leader was synthesized by in vitro transcription, capped and attached to beads via a streptavidin-biotin linkage to incubate with nuclear extract. To exclude abundant RNA-binding proteins that recognize many RNAs, a nascent leader sequence lacking UG repeats from the *CG13089* gene was used as a control. *CG13089* is transcribed in both Sg4 and BG3 cells, but does not bind cohesin in either cell type. (**B**) Silver-stained SDS-PAGE gel separation of proteins retained by leader RNA from the *CG8177* cohesin-binding gene and the *CG13089* gene that doesn’t bind cohesin. Three protein bands that appeared to be bound by the *CG8177* leader RNA but not by the *CG13089* leader were cut out of the gel to be analyzed by mass spectrometry. One of the bands did not contain RNA-binding proteins in either lane. One contained Rm62 in both the *CG8177* and *CG13089* lanes and Rb97D only in the *CG8177* lane. Rb97D is only essential for spermatogenesis [50] and was not further studied. The third band contained Lark (red arrow) an essential RNA-binding protein [51] only in the *CG8177* lane. (**C**) Lark preferentially binds RNAs produced by cohesin-binding genes. The left pie chart shows the fraction of all active genes that bind cohesin (red) and those that don’t (blue) in BG3 cells. Active genes were identified by precision run-on sequencing (PRO-seq [17]) and cohesin binding was determined by ChIP-seq for Rad21 (average of two biological replicates). Active genes were defined as those having ≥100 PRO-seq reads in the 500 nt surrounding the transcription start site, and cohesin binding was defined as enrichment in the 95^th^ percentile for ≥150 bp overlapping the gene. The pie chart on the right shows those genes whose RNAs co-precipitate with Lark [27] and are active in BG3 cells, with the fractions that bind (red) or don’t bind (blue) cohesin. The p value for selective binding of Lark to RNAs from cohesin binding genes is 2.5E-58 by Fisher’s exact test. (**D**) Lark is located primarily in the nucleus in BG3 cells. Fluorescent immunostaining in Mock and Lark-depleted BG3 cells with HP1 heterochromatin protein and Lark [51] antibodies, demonstrating nuclear localization of Lark. See S1 Fig for primers used to prepare double-stranded RNA for Lark depletion and western blots of Lark-depleted cells.
(TIF)

S4 Fig Method used to quantify ChIP-seq enrichment at individual gene regulatory sequences. All gene regulatory sequences were defined as 500 bp elements as illustrated in Fig 2. The browser view shows the example of quantifying Ser5P Pol II ChIP enrichment at the two promoters (pink arrows) of the *string* (*stg*, *cdc25*) gene. The bottom track shows the minus strand PRO-seq used to define the active promoters. The two tracks just above show the coverage at each base pair (number of times each base pair is present in an aligned read) from sequencing input chromatin and the Ser5P Pol II ChIP-seq for one biological replicate. The three tracks above the base pair coverage tracks show the ChIP-seq enrichment of sequences relative to input at 50 bp intervals calculated using sliding 250 bp windows as described elsewhere [24] for two independent biological replicates and the average of the two replicates. The replicate average values were used to calculate the genome-wide Pearson correlation coefficients between ChIP-seq for different proteins given in Fig 2 and S5 Fig. The average of the ten enrichment values (replicate averages) that fall within each of the two promoters is calculated to generate an average log2 enrichment value for each promoter. These average log2 enrichment values for promoters, enhancers, PREs, gene bodies, and random 500 bp segments were used to generate the box and dot plots in Figs 2, 3, 4, 5 and 6 and S6 Fig.
(TIF)

S5 Fig ChIP-seq for Nipped-B, TBPH and Lark in 3^rd^ instar larval wing discs. The genome browser tracks show the log2 ChIP-seq enrichment for Lark (brown) TBPH (orange) and Nipped-B (purple) at the *string* and *Wrinkled* genes. The table shows the genome-wide Pearson correlation coefficients for ChIP-seq enrichment between Nipped-B, TBPH and Lark in 3^rd^ instar wing discs.
(TIF)

S6 Fig Effects of TBPH and Lark depletion on Nipped-B and cohesin (SA subunit) occupancy at active promoters, extragenic enhancers, and PREs in BG3 cells. Each dot is an individual promoter, enhancer or PRE, with its average log2 enrichment in control cells (log2 enrichment mock, x-axis) plotted against its average log2 enrichment in cells depleted for TBPH (iTBPH) or Lark (iLark) (log2 enrichment RNAi, y-axis). The red line has a slope of 1 and an intercept of 0. Dots above the line show an increase in enrichment with protein depletion and dots below the line show a decrease.
(TIF)

S7 Fig Effect of Lark depletion on Nipped-B and SA distribution at the *headcase* gene in BG3 cells. The *headcase* gene has several enhancers (red boxes) upstream of the promoter (pink arrow). The tracks show log2 ChIP-seq enrichment for Nipped-B (purple) and SA (green) in control cells and cells depleted for Lark (iLark). The Lark log2 ChIP-seq enrichment track in control cells is shown in brown. The heat maps below the Nipped-B and SA ChIP-seq tracks show where enrichment decreases (blue) and increases (red) are in the 5^th^ and 95^th^ percentile over regions of 300 bp.
(TIF)

S8 Fig Triptolide treatment does not alter the levels of Nipped-B, TBPH and Lark. The two western blots show the levels of the Nipped-B, TBPH and Lark proteins after treatment of BG3 cells with 10 μM triptolide for up to 4 hours.
(TIF)

S9 Fig Effects of triptolide treatment on Ser5P Pol II, Nipped-B, TBPH and Lark ChIP-seq enrichment at *string* proximal and distal promoters in BG3 cells. The top graph shows the change in Ser5P Pol II and Nipped-B ChIP-seq enrichment relative to the starting level over a 4 hour time course of triptolide treatment at the two *string* promoters. The bottom graph shows the TBPH and Lark enrichment relative to the starting level over the same time course.
(TIF)

S10 Fig Expression of TBPH and Lark full length and truncated proteins in *E*. *coli*. (**A**) Coomassie-stained SDS-PAGE separation of the His_6_-SUMO-TBPH proteins bound to NTA-Zn^2+^ agarose beads used for in vitro RNA (Fig 7, S1 Table) protein-binding (Fig 8) and DNA-binding (S10 Fig) experiments. Diagrams below show the amino acid residues contained in each fragment. (**B**) Coomassie stained SDS-PAGE separation of the His_6_-SUMO-Lark proteins used for in vitro RNA and protein binding experiments. Diagrams below show the amino acid residues contained in each fragment.
(TIF)

S11 Fig Binding experiments with TBPH and Lark and DNA fragments from the *cut* and *Wrinkled* enhancer regions and correlations between TBPH and Lark ChIP-seq enrichment and single-stranded DNA measured by genome-wide KMnO_4_ footprinting in BG3 cells. The two genome browser panels at the top show the active enhancers for *cut* and *Wrinkled* in BG3 cells (red boxes) and DNA fragments (blue boxes) tested for binding to TBPH and Lark on beads (S9 Fig) using the procedure used for RNA-binding (Fig 7) and the log2 ChIP-seq enrichment for Nipped-B, TBPH and Lark. The bar graph below the browser panels show the enrichment of each fragment relative to the W10 DNA fragment. Several fragments show weak enrichment of 2 to 6-fold with Lark beads, which is substantially lower than enrichments seen with RNA (40 to 170-fold) and which do not correlate with the ChIP-seq enrichment. For instance, W1 and W8 show the highest enrichment but are located in regions with little or no Lark enrichment by ChIP-seq. The boxplot at the lower left shows the log2 number of sequence reads in the genome-wide KMnO_4_ footprinting of BG3 cells [36] (GEO accession no. GSE46620) in active promoters (PRO) gene bodies (BOD) extragenic enhancers (ENH) PREs (PRE) and random positions (RAN) defined as shown in Fig 2. The table shows the genome-wide Pearson correlation coefficients between the Ser5P Pol II, Nipped-B, TBPH and Lark ChIP-seq enrichment and KMnO_4_ footprinting reads at promoters, gene bodies, enhancers and PREs.
(TIF)

S12 Fig Co-immunoprecipitation experiments with Nipped-B, TBPH, Lark antibodies and BG3 cell nuclear extract. (**A**) Western blots of protein co-immunoprecipitation of TBPH from BG3 cell nuclear extract with Nipped-B antibodies, and Nipped-B with Lark antibodies. The blot on the left shows TBPH precipitated by immunoprecipitation of Nipped-B in the presence and absence of ethidium bromide, and is representative of three independent experiments. The blot on the right shows precipitation of Nipped-B from BG3 nuclear extract with both Lark and Nipped-B antiserum, and is representative of two independent experiments. (**B**) Western blots of immunoprecipitation of BG3 cell nuclear extract with TBPH and Lark antibodies. The blot on the left shows Lark precipitation by Lark, but not by TBPH antibody. The blot on the right shows precipitation of TBPH but not Lark with TBPH antibody. Both blots are representative of two independent experiments. (**C**) Western blots showing Nipped-B binding to TBPH and Lark beads (S10 Fig) after pre-treatment of BG3 cell nuclear extract with RNase A and RNase T1. All panels are from the same exposure of the same western blot, with irrelevant lanes removed and are representative of two independent experiments.
(TIF)

S1 Table RNAs used for in vitro binding experiments with TBPH and Lark. UG repeats are indicated in red and CGUUC sequences are in bold and underlined. The ability of the RNA to bind (>10-fold enrichment) Lark and TBPH is indicated (Yes or No) and whether or not the RNA is produced by a cohesin-binding gene (Yes or No).
(PDF)

S1 File R scripts used to calculate average ChIP-seq enrichment in gene regulatory elements (ave.chip.r) and sum PRO-seq or KMnO_4_ footprinting reads (sum.reads.r) in gene regulatory elements. (TXT)

S2 File BG3 cell active promoter bed file. (TXT)

S3 File BG3 cell active gene body bed file. (TXT)

S4 File BG3 cell extragenic enhancer bed file. (TXT)

S5 File BG3 cell PRE bed file. (TXT)

S6 File BG3 cell random site bed file. (TXT)

S7 File Bed file of DNA fragments from *cut* and *Wrinkled* enhancer regions tested for binding to TBPH and Lark. (TXT)
