# Supplementary figures and images for: Drosophila TDP-43 RNA-Binding Protein Facilitates Association of Sister Chromatid Cohesion Proteins with Genes, Enhancers and Polycomb Response Elements
Source: PLoS Genet. 2016 Sep 23;12(9):e1006331. doi: 10.1371/journal.pgen.1006331 (PMC5035082; doi:10.1371/journal.pgen.1006331)

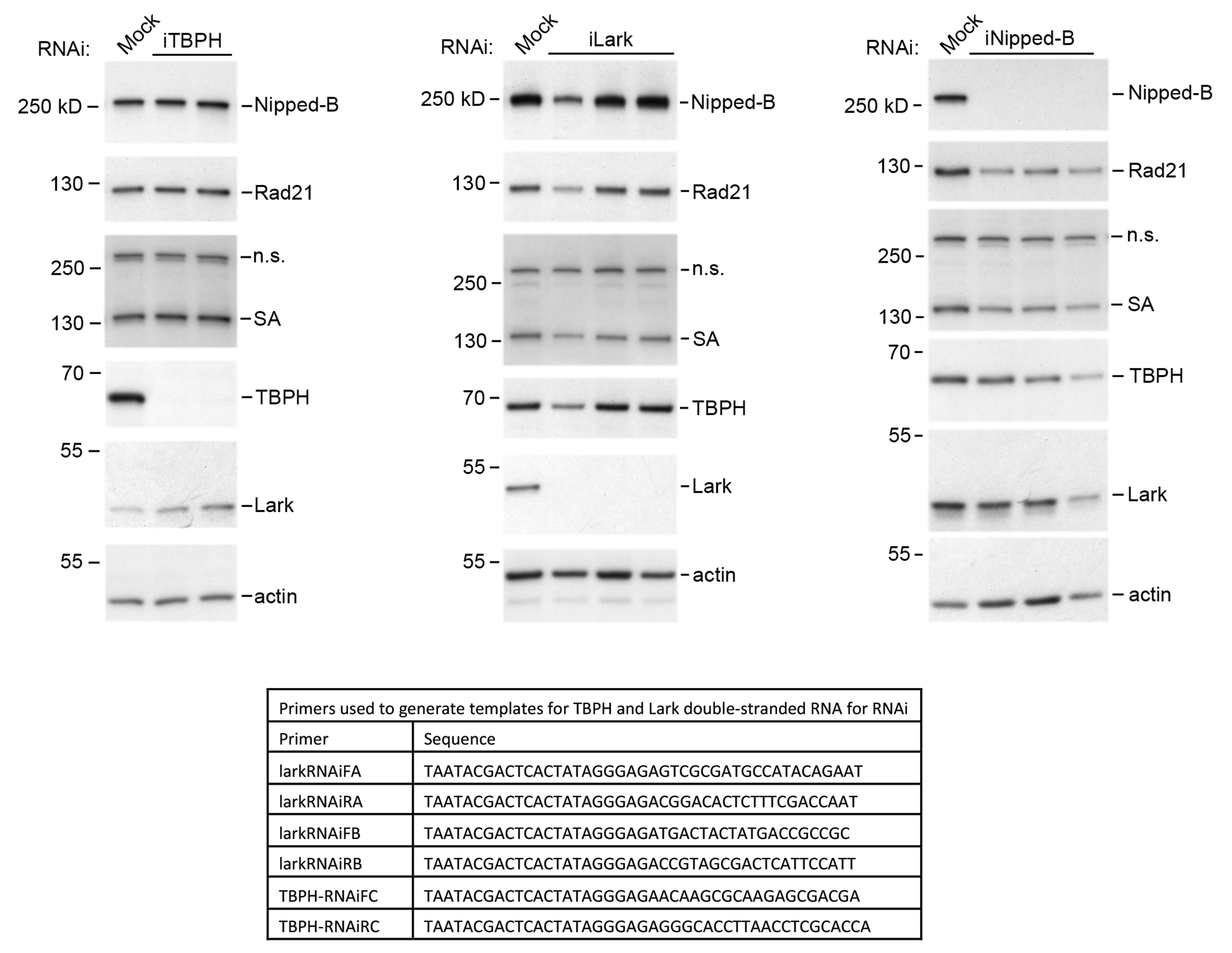

Supplement: S1 Fig — BG3 cells were depleted for the indicated proteins for four to five days and 15 μg of total cell extracts were analyzed by western blotting. The blots shown are representative of at least three independent experiments. The panels on the left compares mock-depleted control cells (Mock) versus cells depleted for TBPH (iTBPH) using double-stranded RNA prepared using the primers in the table at the bottom. The blot was probed with antibodies against Nipped-B, Rad21, SA, TBPH (this study), Lark (this study) and actin antibodies. The panels in the middle compare control cells (Mock) to cells depleted for Lark (iLark) using dsRNA prepared using the primers shown in the table. The right panels compare control cells to cells depleted for Nipped-B (iNipped-B). Western blots with lanes loaded with 7.5 μg or 30 μg of extract gave equivalent results. (TIF) [file pgen.1006331.s001.tif]

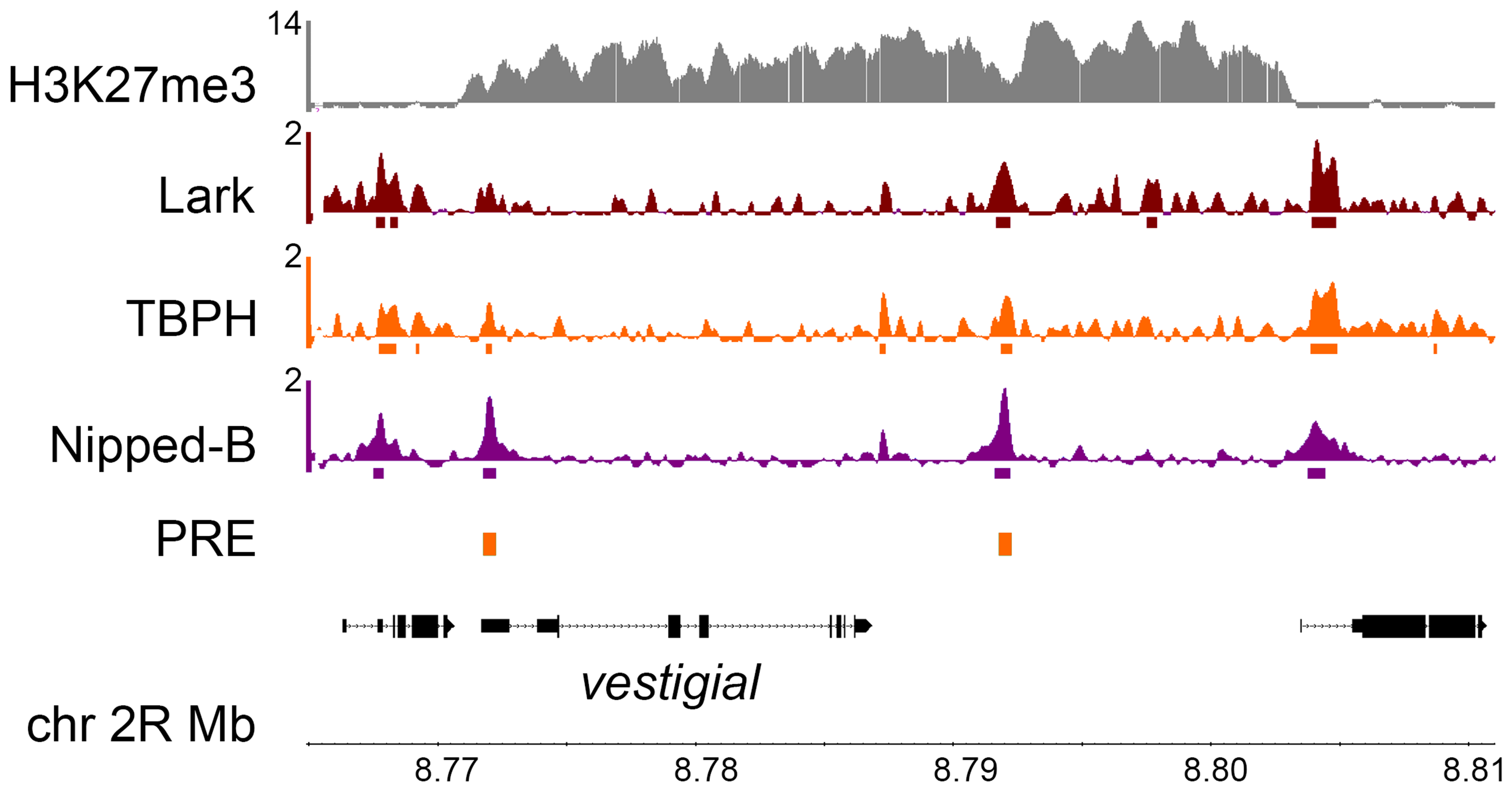

Supplement: S2 Fig — The genome browser view shows the vestigial gene (transcribed left to right) and the PREs (orange boxes) that silence it. The top ChIP-chip track (gray) shows H3K27me3 (histone H3 lysine 27 trimethyl) mark made by the PRC2 silencing complex in BG3 cells [25] with enrichment expressed as MAT [46] score. The brown Lark, orange TBPH and purple Nipped-B ChIP-seq tracks show the log2 enrichment for each protein, and are the average of two to three independent experiments. The bars underneath the ChIP-seq tracks indicate where enrichment is in the 95th percentile for ≥150 bp. (TIF) [file pgen.1006331.s002.tif]

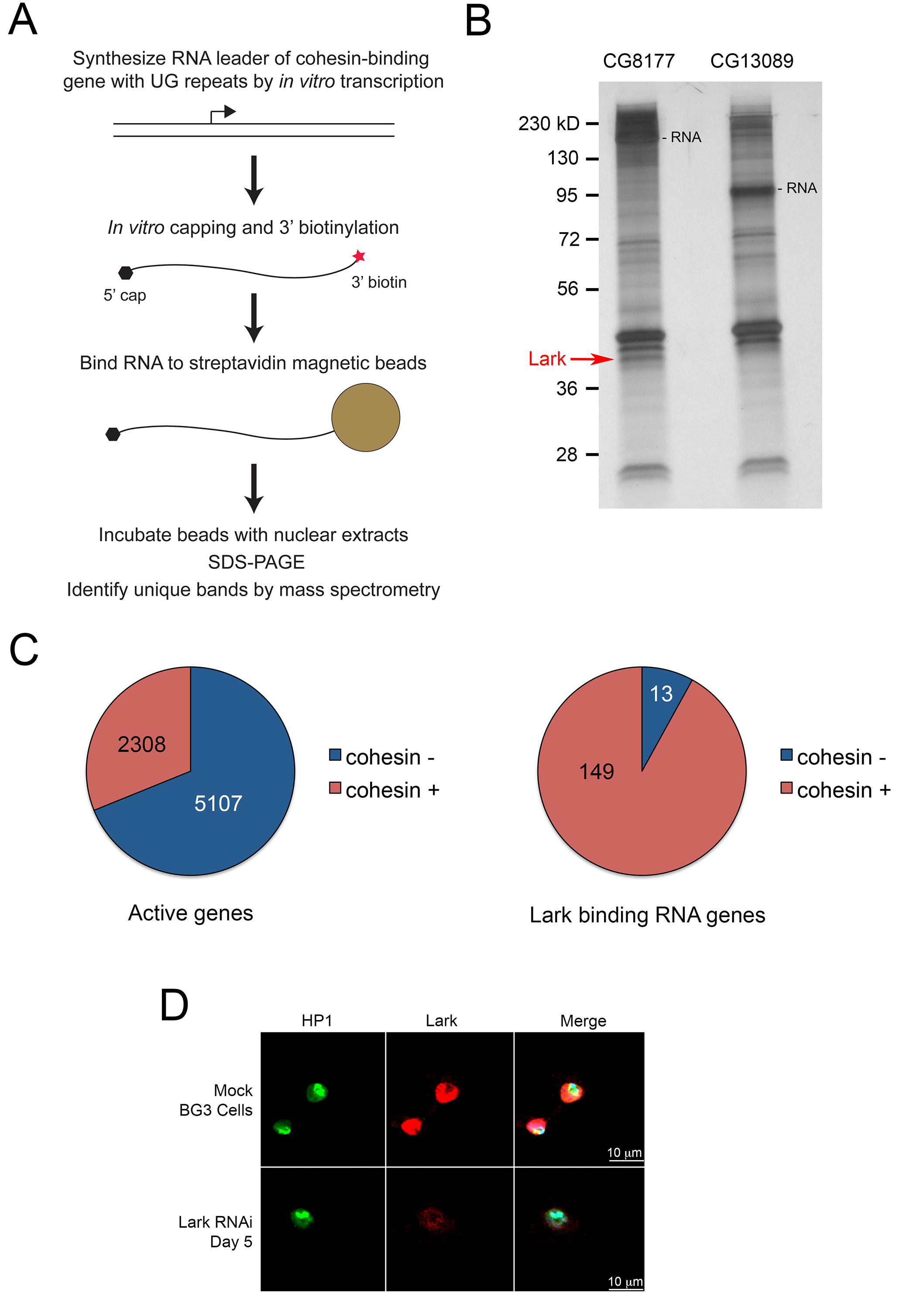

Supplement: S3 Fig — (A) Affinity chromatography strategy to identify RNA binding proteins in nuclear extracts that recognize a nascent RNA leader from a cohesin-binding gene. Nuclear extracts were prepared from cultured ML-DmBG3 (BG3) cells derived from larval central nervous system. The N terminal nascent RNA sequence from the CG8177 gene contains a region with four UG repeats, and one with eight. CG8177 is transcribed and binds Nipped-B and cohesin in both Sg4 (Schneider Line 2 derivative) and BG3 cells [23]. The leader was synthesized by in vitro transcription, capped and attached to beads via a streptavidin-biotin linkage to incubate with nuclear extract. To exclude abundant RNA-binding proteins that recognize many RNAs, a nascent leader sequence lacking UG repeats from the CG13089 gene was used as a control. CG13089 is transcribed in both Sg4 and BG3 cells, but does not bind cohesin in either cell type. (B) Silver-stained SDS-PAGE gel separation of proteins retained by leader RNA from the CG8177 cohesin-binding gene and the CG13089 gene that doesn’t bind cohesin. Three protein bands that appeared to be bound by the CG8177 leader RNA but not by the CG13089 leader were cut out of the gel to be analyzed by mass spectrometry. One of the bands did not contain RNA-binding proteins in either lane. One contained Rm62 in both the CG8177 and CG13089 lanes and Rb97D only in the CG8177 lane. Rb97D is only essential for spermatogenesis [50] and was not further studied. The third band contained Lark (red arrow) an essential RNA-binding protein [51] only in the CG8177 lane. (C) Lark preferentially binds RNAs produced by cohesin-binding genes. The left pie chart shows the fraction of all active genes that bind cohesin (red) and those that don’t (blue) in BG3 cells. Active genes were identified by precision run-on sequencing (PRO-seq [17]) and cohesin binding was determined by ChIP-seq for Rad21 (average of two biological replicates). Active genes were defined as those having ≥100 PRO-seq reads in [file pgen.1006331.s003.tif]

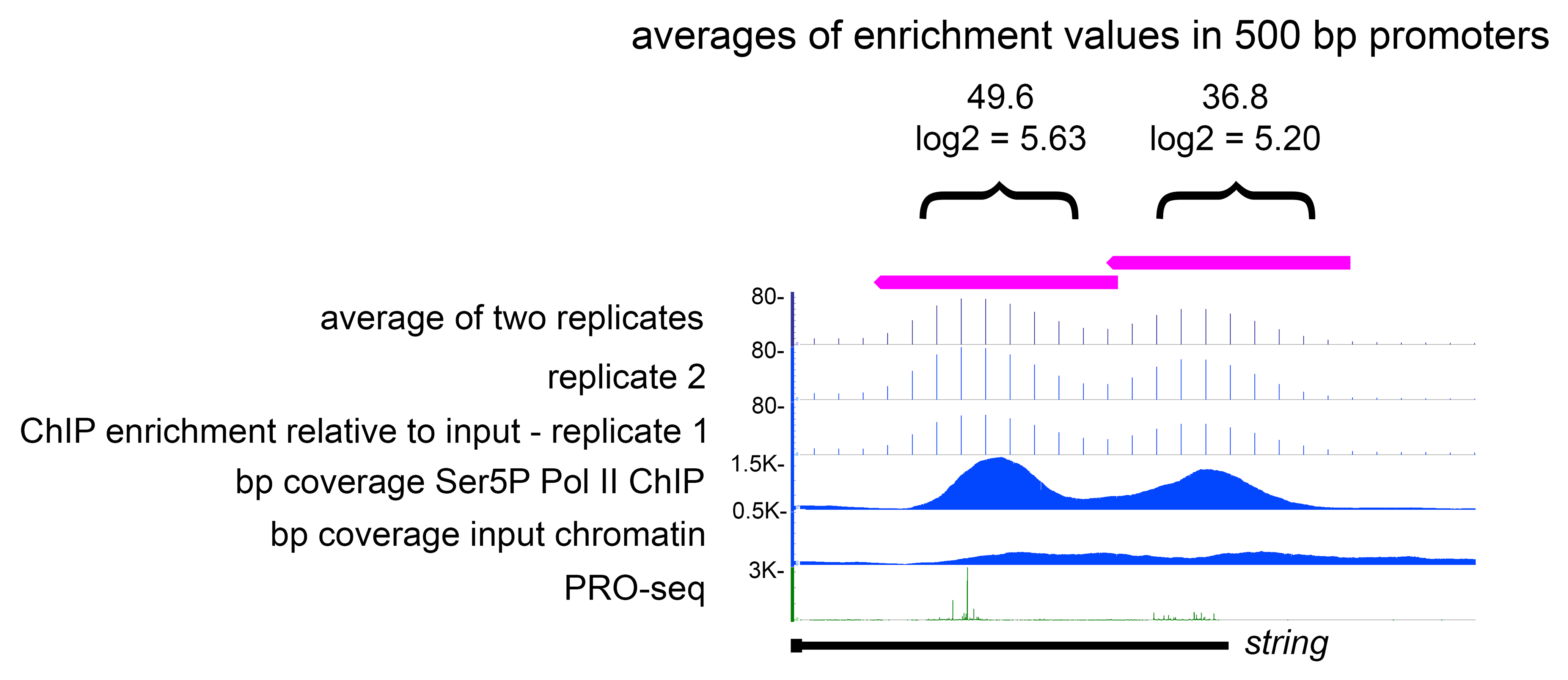

Supplement: S4 Fig — All gene regulatory sequences were defined as 500 bp elements as illustrated in Fig 2. The browser view shows the example of quantifying Ser5P Pol II ChIP enrichment at the two promoters (pink arrows) of the string (stg, cdc25) gene. The bottom track shows the minus strand PRO-seq used to define the active promoters. The two tracks just above show the coverage at each base pair (number of times each base pair is present in an aligned read) from sequencing input chromatin and the Ser5P Pol II ChIP-seq for one biological replicate. The three tracks above the base pair coverage tracks show the ChIP-seq enrichment of sequences relative to input at 50 bp intervals calculated using sliding 250 bp windows as described elsewhere [24] for two independent biological replicates and the average of the two replicates. The replicate average values were used to calculate the genome-wide Pearson correlation coefficients between ChIP-seq for different proteins given in Fig 2 and S5 Fig. The average of the ten enrichment values (replicate averages) that fall within each of the two promoters is calculated to generate an average log2 enrichment value for each promoter. These average log2 enrichment values for promoters, enhancers, PREs, gene bodies, and random 500 bp segments were used to generate the box and dot plots in Figs 2, 3, 4, 5 and 6 and S6 Fig. (TIF) [file pgen.1006331.s004.tif]

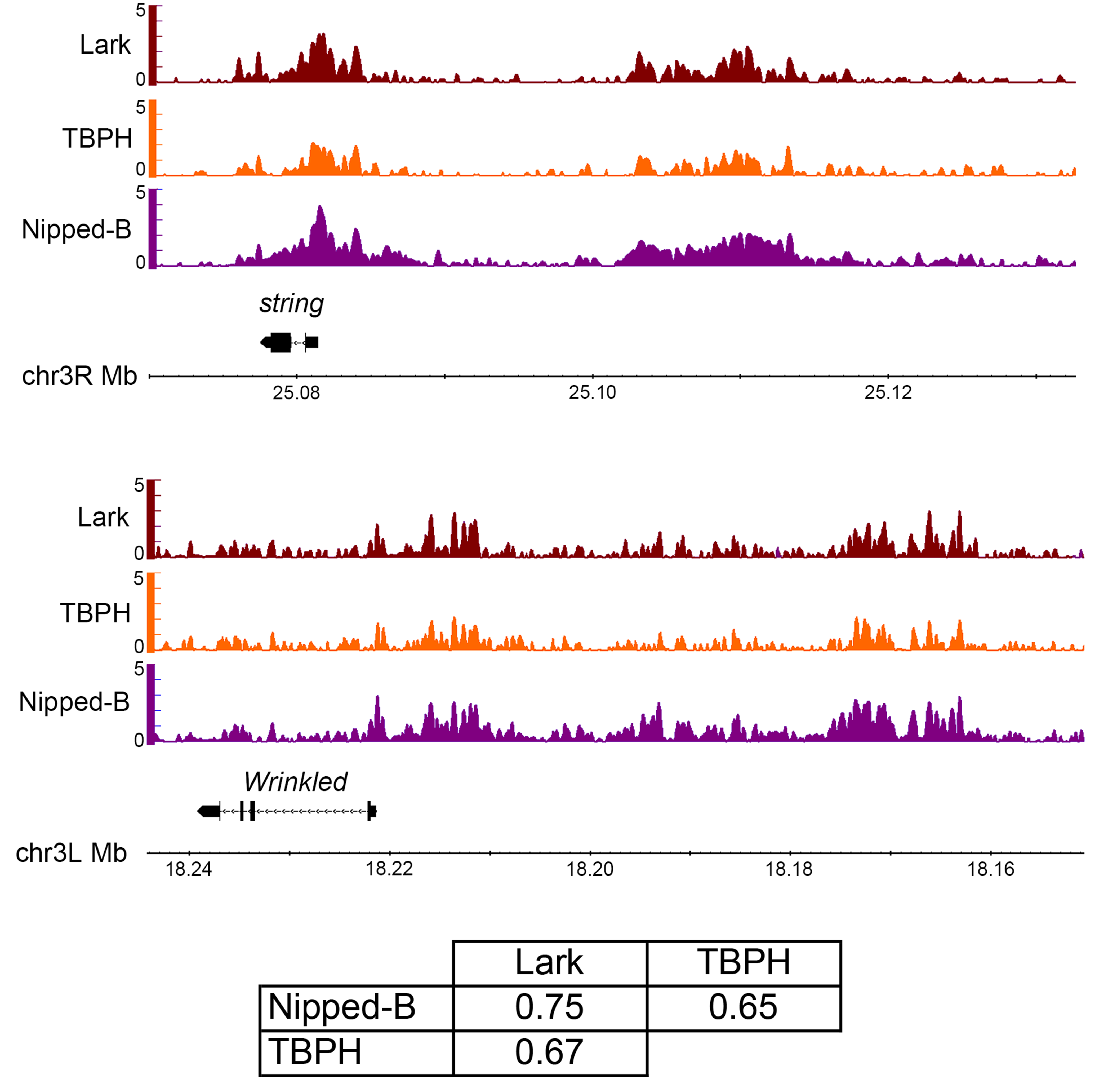

Supplement: S5 Fig — The genome browser tracks show the log2 ChIP-seq enrichment for Lark (brown) TBPH (orange) and Nipped-B (purple) at the string and Wrinkled genes. The table shows the genome-wide Pearson correlation coefficients for ChIP-seq enrichment between Nipped-B, TBPH and Lark in 3rd instar wing discs. (TIF) [file pgen.1006331.s005.tif]

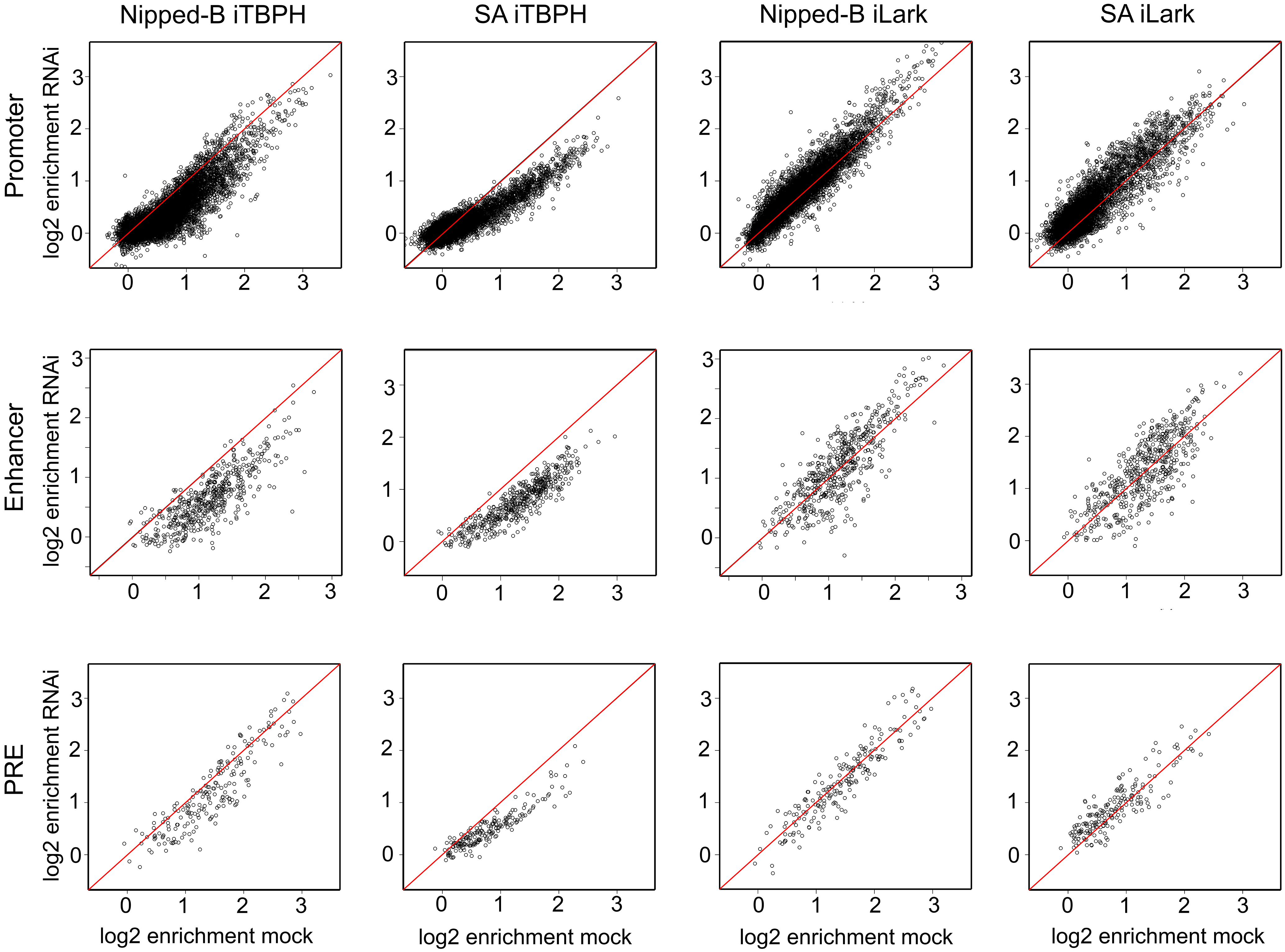

Supplement: S6 Fig — Each dot is an individual promoter, enhancer or PRE, with its average log2 enrichment in control cells (log2 enrichment mock, x-axis) plotted against its average log2 enrichment in cells depleted for TBPH (iTBPH) or Lark (iLark) (log2 enrichment RNAi, y-axis). The red line has a slope of 1 and an intercept of 0. Dots above the line show an increase in enrichment with protein depletion and dots below the line show a decrease. (TIF) [file pgen.1006331.s006.tif]

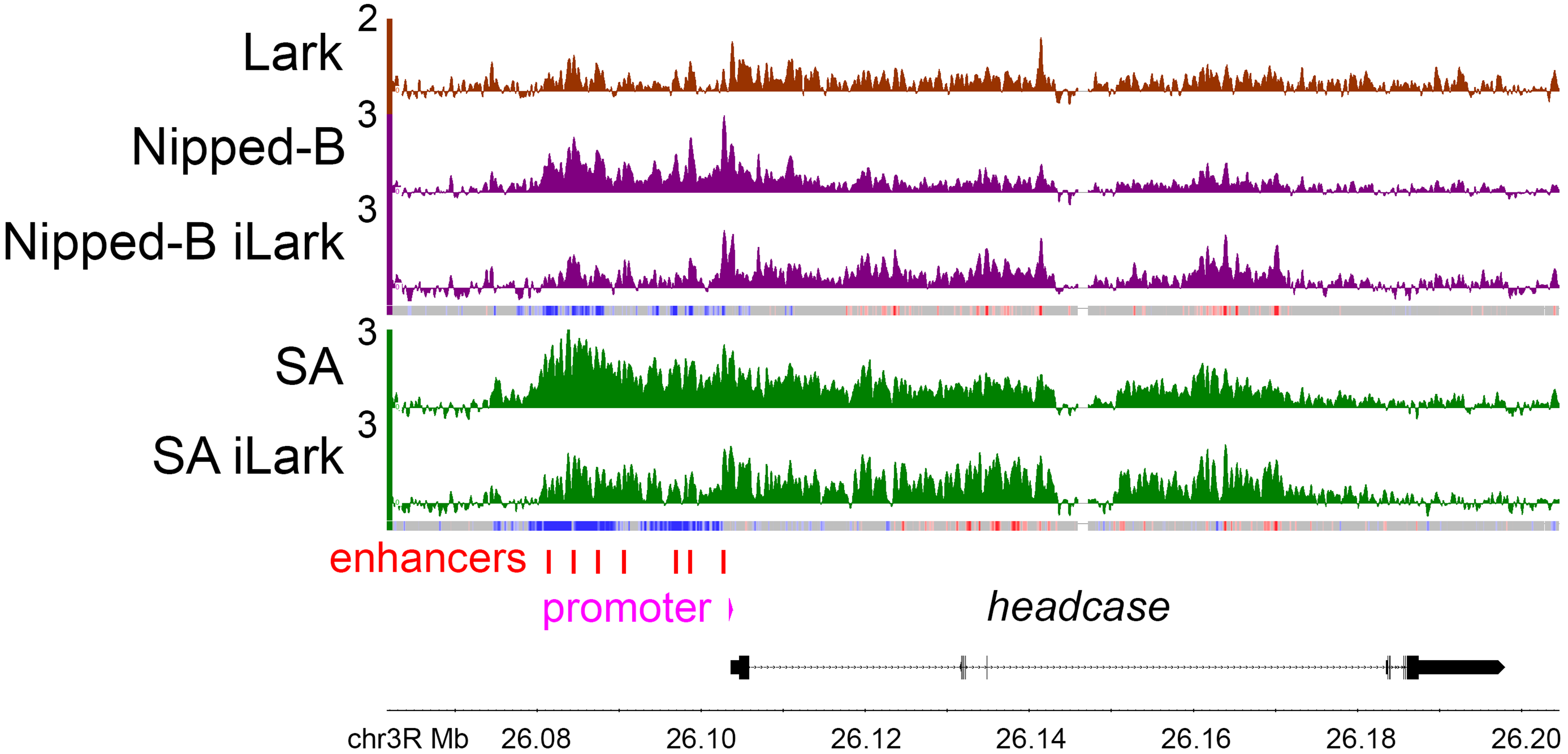

Supplement: S7 Fig — The headcase gene has several enhancers (red boxes) upstream of the promoter (pink arrow). The tracks show log2 ChIP-seq enrichment for Nipped-B (purple) and SA (green) in control cells and cells depleted for Lark (iLark). The Lark log2 ChIP-seq enrichment track in control cells is shown in brown. The heat maps below the Nipped-B and SA ChIP-seq tracks show where enrichment decreases (blue) and increases (red) are in the 5th and 95th percentile over regions of 300 bp. (TIF) [file pgen.1006331.s007.tif]

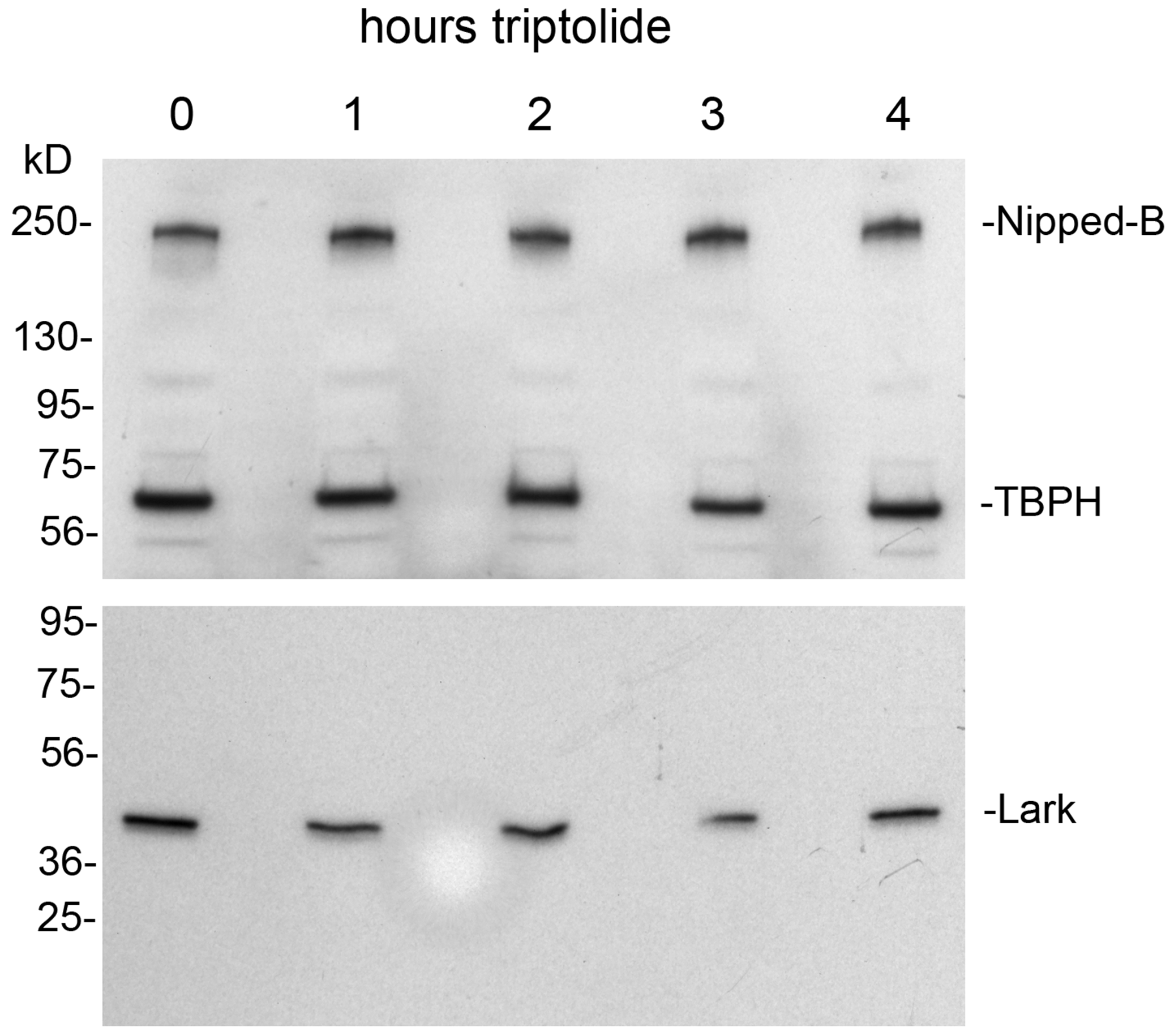

Supplement: S8 Fig — The two western blots show the levels of the Nipped-B, TBPH and Lark proteins after treatment of BG3 cells with 10 μM triptolide for up to 4 hours. (TIF) [file pgen.1006331.s008.tif]

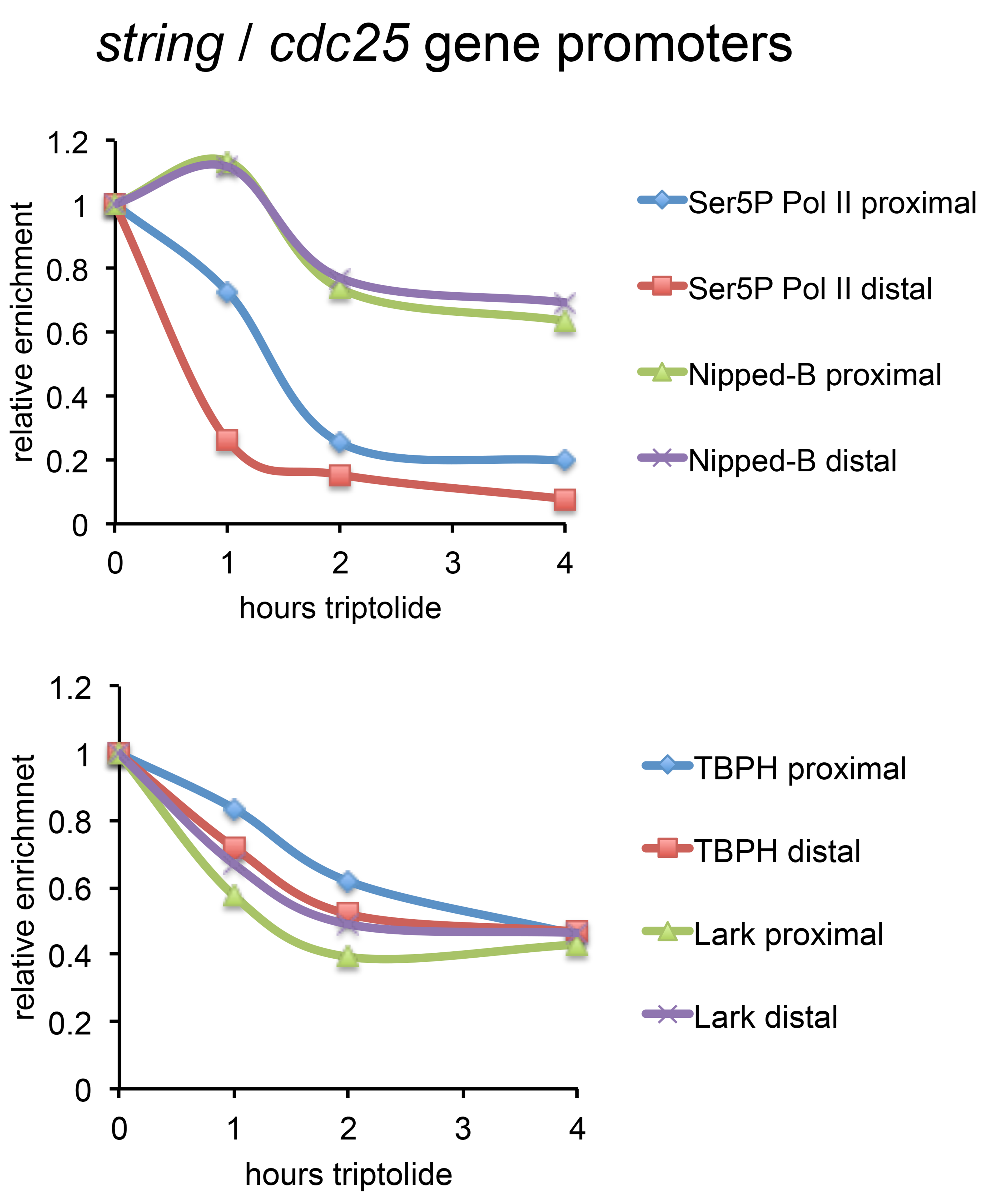

Supplement: S9 Fig — The top graph shows the change in Ser5P Pol II and Nipped-B ChIP-seq enrichment relative to the starting level over a 4 hour time course of triptolide treatment at the two string promoters. The bottom graph shows the TBPH and Lark enrichment relative to the starting level over the same time course. (TIF) [file pgen.1006331.s009.tif]

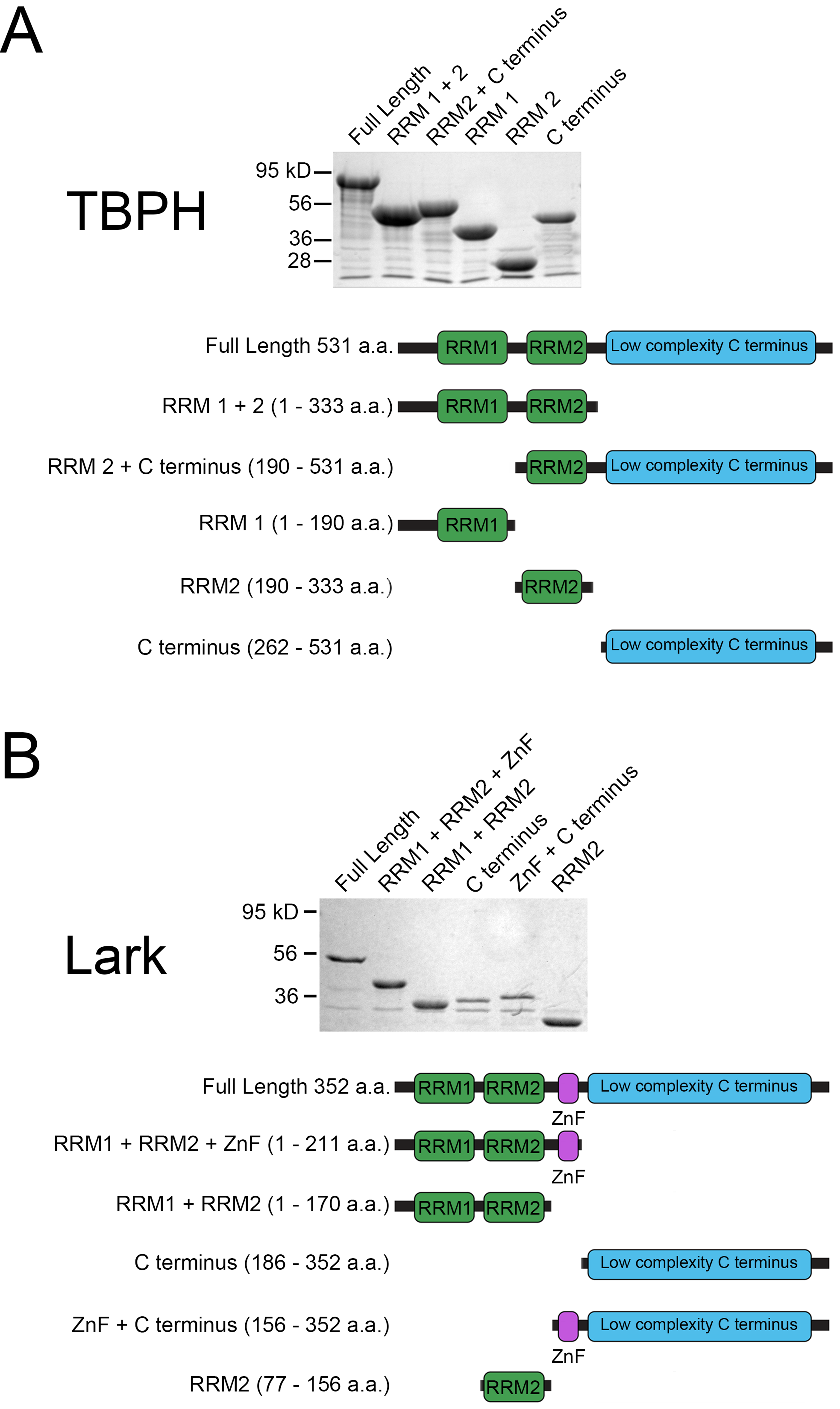

Supplement: S10 Fig — (A) Coomassie-stained SDS-PAGE separation of the His6-SUMO-TBPH proteins bound to NTA-Zn2+ agarose beads used for in vitro RNA (Fig 7, S1 Table) protein-binding (Fig 8) and DNA-binding (S10 Fig) experiments. Diagrams below show the amino acid residues contained in each fragment. (B) Coomassie stained SDS-PAGE separation of the His6-SUMO-Lark proteins used for in vitro RNA and protein binding experiments. Diagrams below show the amino acid residues contained in each fragment. (TIF) [file pgen.1006331.s010.tif]

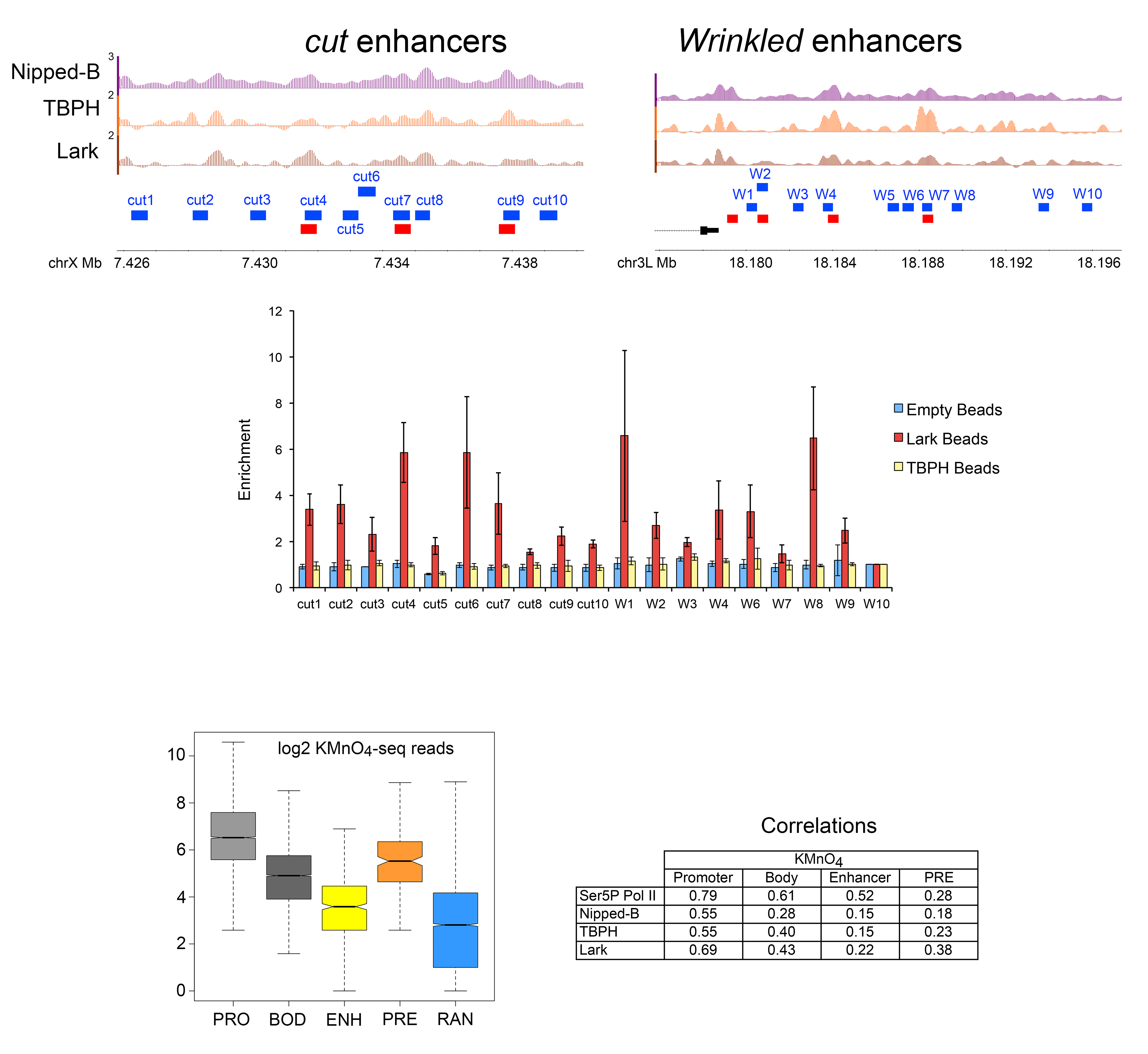

Supplement: S11 Fig — The two genome browser panels at the top show the active enhancers for cut and Wrinkled in BG3 cells (red boxes) and DNA fragments (blue boxes) tested for binding to TBPH and Lark on beads (S9 Fig) using the procedure used for RNA-binding (Fig 7) and the log2 ChIP-seq enrichment for Nipped-B, TBPH and Lark. The bar graph below the browser panels show the enrichment of each fragment relative to the W10 DNA fragment. Several fragments show weak enrichment of 2 to 6-fold with Lark beads, which is substantially lower than enrichments seen with RNA (40 to 170-fold) and which do not correlate with the ChIP-seq enrichment. For instance, W1 and W8 show the highest enrichment but are located in regions with little or no Lark enrichment by ChIP-seq. The boxplot at the lower left shows the log2 number of sequence reads in the genome-wide KMnO4 footprinting of BG3 cells [36] (GEO accession no. GSE46620) in active promoters (PRO) gene bodies (BOD) extragenic enhancers (ENH) PREs (PRE) and random positions (RAN) defined as shown in Fig 2. The table shows the genome-wide Pearson correlation coefficients between the Ser5P Pol II, Nipped-B, TBPH and Lark ChIP-seq enrichment and KMnO4 footprinting reads at promoters, gene bodies, enhancers and PREs. (TIF) [file pgen.1006331.s011.tif]

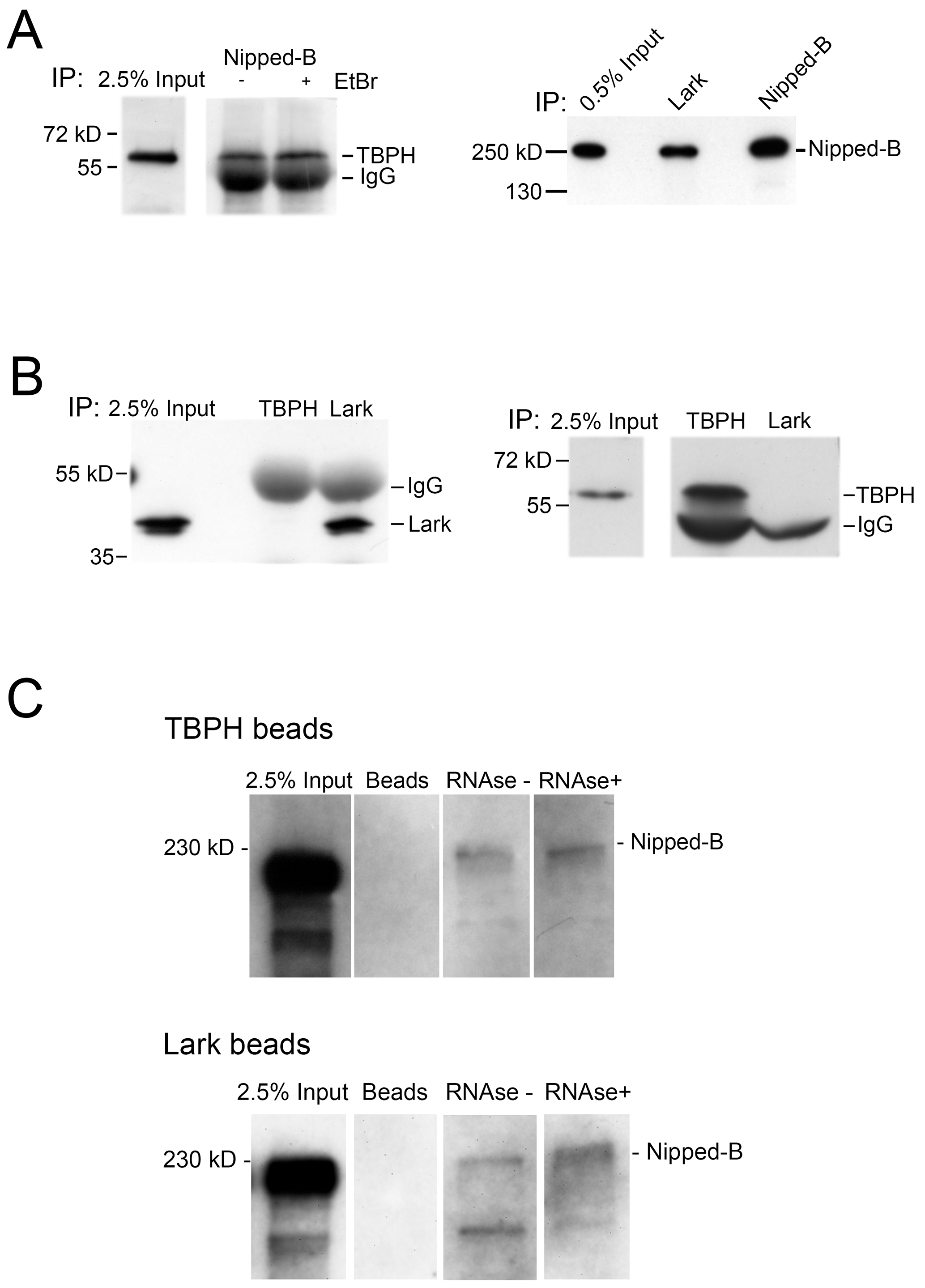

Supplement: S12 Fig — (A) Western blots of protein co-immunoprecipitation of TBPH from BG3 cell nuclear extract with Nipped-B antibodies, and Nipped-B with Lark antibodies. The blot on the left shows TBPH precipitated by immunoprecipitation of Nipped-B in the presence and absence of ethidium bromide, and is representative of three independent experiments. The blot on the right shows precipitation of Nipped-B from BG3 nuclear extract with both Lark and Nipped-B antiserum, and is representative of two independent experiments. (B) Western blots of immunoprecipitation of BG3 cell nuclear extract with TBPH and Lark antibodies. The blot on the left shows Lark precipitation by Lark, but not by TBPH antibody. The blot on the right shows precipitation of TBPH but not Lark with TBPH antibody. Both blots are representative of two independent experiments. (C) Western blots showing Nipped-B binding to TBPH and Lark beads (S10 Fig) after pre-treatment of BG3 cell nuclear extract with RNase A and RNase T1. All panels are from the same exposure of the same western blot, with irrelevant lanes removed and are representative of two independent experiments. (TIF) [file pgen.1006331.s012.tif]
