# Supplementary material for: Drosophila TDP-43 RNA-Binding Protein Facilitates Association of Sister Chromatid Cohesion Proteins with Genes, Enhancers and Polycomb Response Elements
Source: PLoS Genet. 2016 Sep 23;12(9):e1006331. doi: 10.1371/journal.pgen.1006331 (PMC5035082; doi:10.1371/journal.pgen.1006331)
Supplement: S1 Table — UG repeats are indicated in red and CGUUC sequences are in bold and underlined. The ability of the RNA to bind (>10-fold enrichment) Lark and TBPH is indicated (Yes or No) and whether or not the RNA is produced by a cohesin-binding gene (Yes or No). (PDF) [file pgen.1006331.s013.pdf]

Name	Sequence	Lark Binding	TBPH Binding	Binds cohesin
**CG8177-1**	GGAACUGUAAAUCAUUA**CGUUC**GGAAACGUGC<br>UGCUGCUCGGACGGAAACGCAGAAACUG	Yes	No	Yes
**CG8177-2**	ACUGCGGAUAAAAGGCGGACAAUAUUUUAGA<br>GCUCGCCAAGUUCAAGUUUGCGA	No	No	Yes
**CG8177-3**	UCGCCAAGUUCAAGUUUGCGACGAAACACAGA<br>AAGUGGUAAACCGUACGCG	No	No	Yes
**CG8177-4**	AUAUACAAAAGGUUCCACCACCAACGUGCGCA<br>CACGCAACAAUAGCAACA	No	Yes	Yes
**CG8177-5**	GCACACGCAACAAUAGCAACAACAACAAAAAAA<br>AAAGCGCCGUUGUUAUUGUUGCU	No	No	Yes
**CG8177-6**	CGCCGUUGUUAUUGUUGCUCUUUCCUGCCAA<br>UUUUAUUGUGUUUCUCCAUUGCCCUCUGC	No	No	Yes
**CG8177-7**	CCCUCUGCUUUAUCGUCUGUUGUUUUUAUCG<br>ACG**CGUUC**GCUCCCG**CGUUC**UUUCUCCUU	Yes	No	Yes
**CG8177-8**	CG**CGUUC**UUUCUCCUUCUCCAGCCAGCGCGA<br>GUGUGUGUGUGUGUGUGUCUCG	Yes	Yes	Yes
**CG8177-9**	AUAUACAAAAGGUUCCACCACCAACGUGCGCA<br>CACGCAACAAUAGCAACAACAACAAAAAAAAAA<br>GCGCCGUUGUUAUUGUUGCUCU	No	No	Yes
**CG6310-1**	GCCCUGCUCCUCUAUGUCUUCGUUUUCUCCA<br>GUGGCUATCAGAAGUACCAGAUCGAGGGC	No	No	No
**CG6310-2**	CGAGGGCAUUAUCAAGCAGAGUCAACCGGAAA<br>AGGUCUGGGAGUAUGUGGCGGAUU	No	No	No
**CG6310-3**	AAGGUCUGGGAGUAUGUGGCGGAUUUUAAUA<br>AGAUGCGUCUGCTCAAUCCCACCAU	No	No	No
**CG6310-4**	CCUAGCUGAUCAUGGCCACGCCCACGACUGG<br>CGUUACACGGUGCAGUACAC	No	No	No
**CG6310-5**	UGGUCACCAAGUCAUUACCCGGAGUAUCGCC<br>CGUGGCCUAUGCCAUUAAGU	No	No	No
**CG6310-6**	A**CGUUC**AGAAACUCCAAUGGUGAUACCUUUGC<br>CCAGGAGAACAUCCAGUA	No	No	No
**CG13089-1**	GCUUUUUACAGGCGGAGUC**CGUUC**ACAAAAAC<br>UAUGCCCAACUAGAAGAUGGAUUCG	No	No	No
**cut-1**	CAGCAUUCGUGACUGGCAACGUCGUUU**CGUU**<br>**C**GUCCGUCGCCGAAAGAAAUCGAGA	Yes	No	Yes
**Notch-1**	AGUGGUGUACAGUGGUCACUCAUUCAUAUUG<br>UAUGUGUGUGUGUGUGUGUGUAUCGG**CGUUC**	No	Yes	Yes
**trol-1**	UUUGUGCCUGAAGGAGGGAGGGGUCGCUGUG<br>UGUGUGUGUGCGAGUGUGUGUGUGUGUAUG	Yes	Yes	Yes
**trol-2**	UGACUCUUGGACCAAAAGCCAAACUUGAUUUA<br>UUUCCUGUUUCUGUGUGUGUGUGCGUGUUG	No	Yes	Yes
**rho-1**	GCGACUCGAAACUGAAAUUGAAACUGAAAGAA<br>AGAAAUAUUCAAAUUGUCGUGUGUGUGG	Yes	NT	Yes
**rho-2**	CACAAAAGCAACAACGGUGCGUGUGUGCAAUG<br>CGACUUGAGG	Yes	NT	Yes
**rho-3**	CCCAGAAUUUCGGGCUACC**CGUUC**CGAUUCC<br>GUCGGAUUCGGUGCUGGUCUAU	No	NT	Yes
**path-1**	AAGUUCCAGUUUGAAUGCCGCUUGAAU**CGUU**<br>**C**AGAACUGU	No	NT	Yes
**path-2**	AAAACAAGAGCGCCAAUGCCGCACAACGUGAU<br>GAUUG**CGUUC**UUCCACAGGAUCCA	No	NT	Yes
**path-3**	CUGC**CGUUC**CCGUCCAAUAGGAAAUCAACUGC<br>UCAAAGUUCGAGGCCA	No	NT	Yes
**path-4**	GUGGCUUUGGGAUUGAUUUGCUUCAAUUG**CG**<br>**UUC**UAUACGAUCGCUAUUUACAUUGGGCC	Yes	NT	Yes
**path-5**	CGCGAGUGUACUAAAACGUGCUAAAACGGCAU<br>AAUGGUGCUCGGUAGUGUGCUAC	Yes	NT	Yes
**path-6**	GUGGGGCAGUUGCAUUGACGUGUGUGUGCAG<br>ACAGCUAUG	Yes	NT	Yes
**path-7**	UCGGCCAUGUCCUUGAUGACUCAGUUCGAAU<br>CGGUCUAAAAGUGUGUGGGCGUGG	No	NT	Yes
